# G6PD maintains the VSMC synthetic phenotype and accelerates vascular neointimal hyperplasia by inhibiting the VDAC1–Bax-mediated mitochondrial apoptosis pathway

**DOI:** 10.1186/s11658-024-00566-w

**Published:** 2024-04-08

**Authors:** Ting Zhang, Rui-Jie Cao, Jiang-Ling Niu, Zhi-Huan Chen, Shi-Qing Mu, Tong Cao, Jie-Xin Pang, Li-Hua Dong

**Affiliations:** 1 Department of Biochemistry and Molecular Biology, College of Basic Medicine, Cardiovascular Medical Science Center, Key Laboratory of Vascular Biology of Hebei Province, Key Laboratory of Neural and Vascular Biology of Ministry of Education, Hebei Medical University, Shijiazhuang, 050017 China; 2 Department of Nuclear Medicine, The Fourth Hospital of Hebei Medical University, Shijiazhuang, 050011 China

**Keywords:** Vascular smooth muscle cells, Apoptosis, G6PD, VDAC1, Bax

## Abstract

**Background:**

Glucose-6-phosphate dehydrogenase (G6PD) plays an important role in vascular smooth muscle cell (VSMC) phenotypic switching, which is an early pathogenic event in various vascular remodeling diseases (VRDs). However, the underlying mechanism is not fully understood.

**Methods:**

An IP‒LC‒MS/MS assay was conducted to identify new binding partners of G6PD involved in the regulation of VSMC phenotypic switching under platelet-derived growth factor-BB (PDGF-BB) stimulation. Co-IP, GST pull-down, and immunofluorescence colocalization were employed to clarify the interaction between G6PD and voltage-dependent anion-selective channel protein 1 (VDAC1). The molecular mechanisms involved were elucidated by examining the interaction between VDAC1 and apoptosis-related biomarkers, as well as the oligomerization state of VDAC1.

**Results:**

The G6PD level was significantly elevated and positively correlated with the synthetic characteristics of VSMCs induced by PDGF-BB. We identified VDAC1 as a novel G6PD-interacting molecule essential for apoptosis. Specifically, the G6PD-NTD region was found to predominantly contribute to this interaction. G6PD promotes VSMC survival and accelerates vascular neointimal hyperplasia by inhibiting VSMC apoptosis. Mechanistically, G6PD interacts with VDAC1 upon stimulation with PDGF-BB. By competing with Bax for VDAC1 binding, G6PD reduces VDAC1 oligomerization and counteracts VDAC1–Bax-mediated apoptosis, thereby accelerating neointimal hyperplasia.

**Conclusion:**

Our study showed that the G6PD–VDAC1–Bax axis is a vital switch in VSMC apoptosis and is essential for VSMC phenotypic switching and neointimal hyperplasia, providing mechanistic insight into early VRDs.

**Graphical Abstract:**

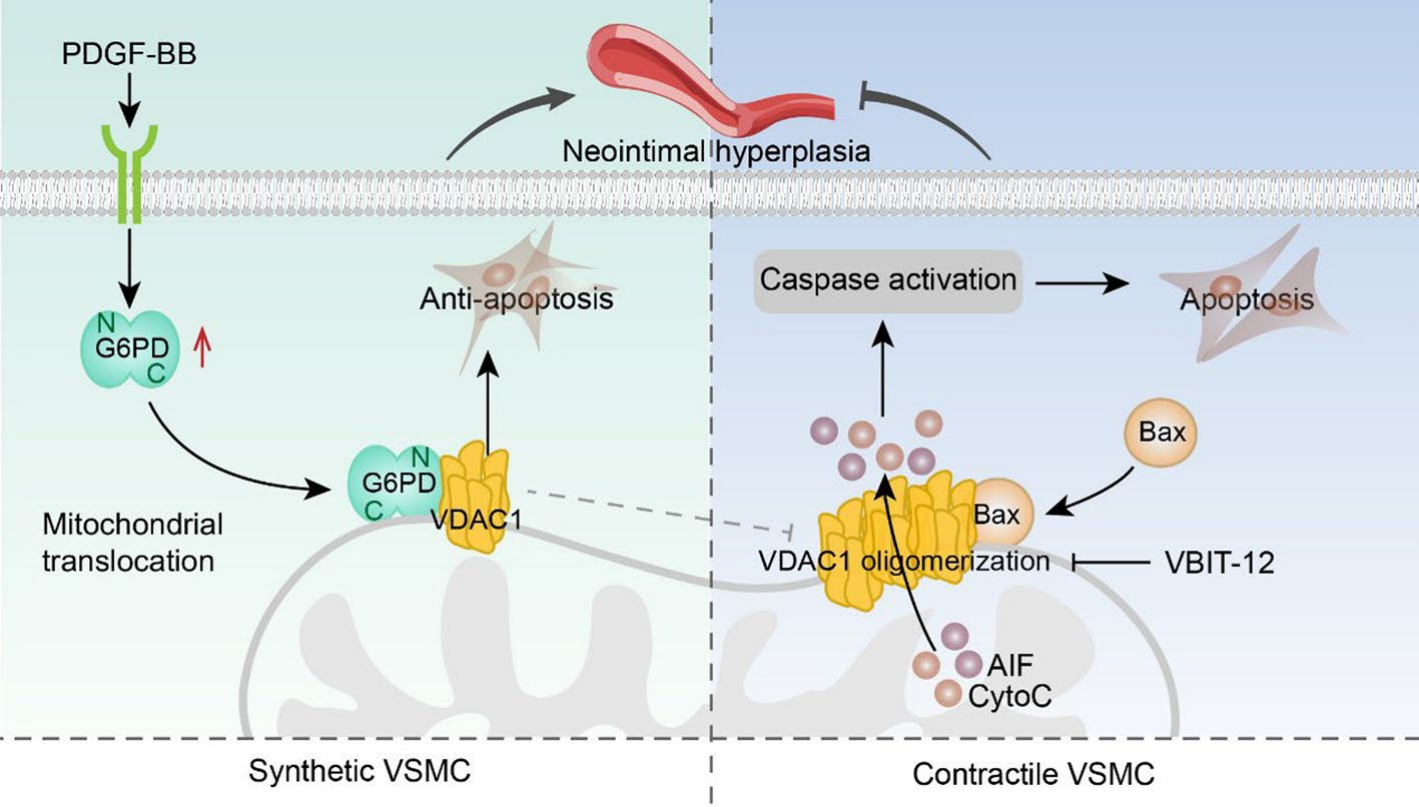

**Supplementary Information:**

The online version contains supplementary material available at 10.1186/s11658-024-00566-w.

## Background

Vascular smooth muscle cells (VSMCs) residing in the tunica media layer of the blood vessel wall are key vascular cells for the control of vascular tone through vasoconstriction [1]. VSMCs demonstrate remarkable plasticity, and platelet-derived growth factor-BB (PDGF-BB) is considered to be the most potent stimulant for VSMC phenotypic switching [2]. The dynamic switching of the VSMC phenotype from contractile to synthetic is a common pathogenesis of various vascular remodeling diseases (VRDs) [3–5] and is typically characterized by a loss of contractile function, replacement of proproliferative and antiapoptotic factors, and increased secretion of extracellular matrix [6–9]. In particular, when vascular endothelial damage or postoperative restenosis occurs, an imbalance between excessive VSMC proliferation and insufficient apoptosis may lead to the abnormal accumulation of vascular intimal cells, which can cause atherosclerosis or restenosis [10–12]. A classic rat model for studying vascular injury has shown that the synthetic peptide MRSP effectively inhibits neointimal hyperplasia by promoting VSMC apoptosis [13]. A previous study demonstrated that miR-637 suppressed the proliferation and migration of VSMCs while promoting apoptosis, thus contributes to atherosclerosis inhibition [14]. By targeting the EPAS1/SLC3A2 pathway, HCMV-miR-US33-5p suppressed the proliferation and promoted the apoptosis of aortic VSMCs in patients with acute aortic dissection (AAD) [15]. Synthetic VSMCs possess proproliferative and antiapoptotic traits that enhance their survival and directly lead to intimal thickening, ultimately worsening the progression of VRDs [16, 17]. However, the mechanism leading to the resistance of synthetic VSMCs to apoptosis has not been fully elucidated.

Glucose-6-phosphate dehydrogenase (G6PD) is the rate-limiting enzyme of the pentose phosphate pathway (PPP) [18]. NADPH produced in the oxidative phase of the PPP (oxPPP) and ribulose-5-phosphate (R5P) produced in the nonoxPPP not only provide the basis for the synthesis of fatty acids, cholesterol, and nucleotides but also help cells resist oxidative stress by scavenging reactive oxygen species (ROS) and maintaining the reduced state of glutathione (GSH) [19]. In this scenario, G6PD tends to be highly expressed in rapidly proliferating cancer cells, such as liver cancer cells, triple-negative breast cancer cells, and prostate cancer cells [18, 20–22]. Consistently, a high level of G6PD is observed in proliferative/synthetic VSMCs, which has also been proposed to play a critical role in VSMC phenotypic switching [23]. Subsequent research revealed that G6PD maintains the VSMC contractile phenotype by regulating smooth muscle cell (SMC)-restricted gene expression, thereby maintaining vascular function and preventing vascular dysfunction [24]. In our previous study, we discovered that TRAF6-induced SM22α ubiquitination plays a crucial role in maintaining VSMC survival by enhancing G6PD activity and NADPH production. This pathway, involving TRAF6-SM22α-G6PD, is a novel mechanism that explains the connection between glucose metabolism and VSMC survival [25]. Additionally, G6PD regulates the relaxation and contraction of vascular smooth muscle by modulating the opening and closing of Ca^2+^ or K^+^ channels [26]. Furthermore, NADPH, a metabolite of G6PD, is involved in the regulation of vascular smooth muscle contraction and relaxes vascular smooth muscle by inhibiting the dimerization of PKG1α [27]. Although there is some evidence indicating that G6PD may affect VSMC phenotypic switching, the molecular mechanism involved remains incomplete, and further research is necessary to comprehensively elucidate this association.

Here, to address the knowledge gap regarding the role of G6PD in preventing apoptosis in synthetic VSMCs, we employed immunoprecipitation (IP) and liquid chromatography-tandem mass spectrometry (LC‒MS/MS) techniques to investigate the protein‒protein interaction (PPI) network of G6PD. Our findings revealed that VDAC1 is a novel G6PD interaction partner associated with apoptosis. We then confirmed that G6PD suppresses VSMC apoptosis through direct interaction with VDAC1. This interaction inhibits VDAC1 oligomerization through competitive binding to Bax, indicating a novel epigenetic mechanism for phenotypic switching to proliferative/antiapoptotic VSMCs. These findings also suggest that this interaction could lead to potential biomarkers and therapeutic targets for VRDs.

## Materials and methods

### Cell culture and treatment

VSMCs were isolated from the aortas of 60–80 g male Sprague‒Dawley (SD) rats anesthetized intraperitoneally with urethane and cultured in low-glucose DMEM (Invitrogen, USA) supplemented with 10% FBS (Gibco, USA) at 37 °C in a humidified atmosphere containing 5% CO_2_. Cells at passages 3–5 were used in all of the experiments unless stated otherwise. Human embryonic kidney 293A cells were obtained from ATCC and cultured in high-glucose DMEM (Invitrogen) containing 10% FBS. Before stimulation with platelet-derived growth factor-BB (PDGF-BB) (20 ng/mL; R&D Systems, USA), the VSMCs were incubated in serum-free medium for 24 h.

### Ligation model and adenovirus infection of mouse common carotid arteries

Male C57BL/6J mice (8 weeks old) were obtained from Liaoning Changsheng Biotechnology Company (Liaoning, China). To induce neointimal hyperplasia, the mouse common carotid artery was ligated following previously described methods [28]. The mice were housed in a pathogen-free laboratory with a 12-h light/dark cycle at a temperature of 22 °C, and they had unrestricted access to food and water. The common carotid artery was carefully dissected from the surrounding connective tissue, and Ad-Null or Ad-G6PD was applied around the carotid artery using 20 μL of Pluronic F127 gel (Sigma‒Aldrich, USA). After 14 days of ligation, the carotid arteries were harvested. The frozen arterial segments were sectioned at 5 μm, stained with hematoxylin and eosin (HE) or immunofluorescence (IF), and examined under a light microscope (Nikon, Japan).

### Western blot analysis

Equal amounts of extracts (20 μg) were separated by 10% SDS‒PAGE and transferred to PVDF membranes. After blocking with 5% milk in TBST, the following specific primary antibodies were used: anti-G6PD (1:1000; Abcam, UK), anti-VDAC1 (1:1000; Abcam), anti-Bax (1:2000; Abways, China), anti-Bcl-2 (1:500; Abways), anti-SM22α (1:2000; Abcam), anti-PCNA (1:1000; Abcam), anti-VDAC2 (1:1000; Abcam), anti-VDAC3 (1:500; Wanlei bio, China), anti-β-actin (1:1000; PTM bio, China), anti-α-actin (1:1000; PTM bio), anti-GAPDH (1:1000; Proteintech, China), anti-Tom40 (1:1000; Proteintech), anti-Caspase 9 (1:1000; PTM bio), anti-Cleaved Caspase 9 (1:1000; Wanlei bio), anti-Caspase 7 (1:1000; PTM bio), anti-cleaved Caspase 7 (1:1000; Wanlei bio), anti-Caspase 3/Cleaved Caspase 3 (1:1000; Wanlei bio), and anti-PARP/Cleaved PARP (1:1000; Wanlei bio), and incubated at 4 °C overnight. The blots were visualized using a GE ImageQuant™ LAS 4000 detection system (USA). Band intensities were quantified with ImageJ software (USA).

### Immunofluorescence (IF)

For ex vivo immunofluorescence staining, after being fixed in 4% paraformaldehyde for 15 min, the cells were washed twice in 1 × PBS for 5 min and then permeabilized with 0.5% Triton X-100 (Beyotime, Shanghai, China) at room temperature for 15 min. The cells were blocked with 1% bovine serum albumin for 20 min and then incubated with primary antibody at 4 °C overnight. After washing with PBS three times, the cells were incubated with goat anti-rabbit IgG secondary antibodies (FITC Green goat anti-rabbit; Molecular Probes, Shanghai, China) for 1 h at room temperature. The nucleic acids were stained with DAPI (Sigma‒Aldrich, Shanghai, China) and normal rabbit IgG (1:50; Proteintech) staining was used as a negative control. Confocal microscopy was performed with a confocal laser scanning microscope system (Leica, GER).

For in vivo immunofluorescence analysis, tissue sections (4 μm) were fixed and stained. Briefly, the samples were incubated with antibodies against G6PD (1:50; Abcam), PCNA (1:50; Abcam), Cleave Caspase 3 (1:50; Wanlei bio), Cleave Caspase 9 (1:50; Wanlei bio), MMP2 (1:50; Proteintech), and collagen1 (1:50; Proteintech). DAPI (Sigma‒Aldrich; Shanghai, China) staining was performed to visualize the nuclei, and normal rabbit IgG (1:50; Proteintech) staining was used as a negative control. After incubation with Dylight 488, Goat Anti-Rabbit IgG (1:200, Proteintech), the images were observed using a confocal laser scanning microscope system (Leica). ImageJ software was used to calculate the mean fluorescent intensity (MFI).

### G6PD enzymatic activity assay

G6PD activity was determined according to previous methods [29]. The enzyme activity of G6PD was assayed by a G6PD Activity Assay Kit. The cells were lysed with ice-cold cell lysis buffer supplemented with PMSF. After centrifugation, the supernatant was collected and mixed with the detection solution. The fluorescence intensity was read with excitation at 540 nm and emission at 590 nm to obtain kinetic curves.

### DSS crosslinking assay

DSS crosslinking was performed according to a published protocol [30]. After washing with cold 1 × PBS, the cells were suspended in conjugation buffer (20 mM HEPES, pH 8.0). The DSS solution in DMSO was added to the cell suspension to a final concentration of 5 mM. After incubating at 37 °C for 30 min, the samples were boiled and subjected to western blotting.

### Oxygen consumption rate (OCR) and extracellular acidification rate (ECAR)

Real-time OCR and ECAR measurements were carried out using a Seahorse Bioscience Extracellular Flux Analyzer (XF24) (Agilent, USA) according to the manufacturer’s protocol. To directly assess mitochondrial metabolism, OCR and ECAR were measured before and after the sequential injection of 4 μM oligomycin, 0.25–5 μM FCCP, and 2 μM rotenone/myxothiazol at the time points specified. The OCR and ECAR were normalized to a cell density of 40,000/well in an XF24 plate. The OCR was measured to determine the optimal concentration of FCCP for controlling basal respiration and maximal respiration in VSMCs. All reagents were purchased from Seahorse Biosciences.

### TUNEL assay

For terminal deoxynucleotidyl transferase (TdT) dUTP nick-end labeling (TUNEL) assays, VSMCs were fixed in 4% paraformaldehyde for 15 min. The cells were then stained with a TUNEL Apoptosis Assay Kit (Beyotime, C1088, China) according to the manufacturer’s protocol. Nuclei were stained with 4′,6-diamidino-2-phenylindole (DAPI). Images were acquired with a laser scanning confocal microscope (Leica).

### Cell viability assay

Cell viability was assessed using a Cell Counting Kit-8 (CCK8) assay. The CCK-8 assay was carried out according to the standard protocol by seeding cells in a 96-well plate at a density of 2000 cells per well and measuring at 450 nm. The cells in each well were treated with 10 µL of CCK8 reagent and 100 µL of DMEM (FBS-free).

### Cell counting assay

Vascular smooth muscle cells (VSMCs) were seeded onto 6-cm plates at a density of 200,000 cells per plate. When reaching a cell density of 60–80%, the plates were treated with either 6AN or siRNA for the specified duration, followed by stimulation with PDGF-BB for 12 h. Afterwards, the plates were harvested and the cells were counted to evaluate cell proliferation. Cell counting was performed using a TC20 automated cell counter (Bio-Rad, USA).

### Flow cytometry analysis and apoptosis

For cell apoptosis analysis, the cells were stained with PI and Annexin V-FITC according to the manufacturer’s instructions (BD, 559763, USA). Fluorescence-labeled cells were analyzed using the guava EasyCyte system (Merck, USA), and FlowJo V10 software was used for data analysis and quantification.

### RNA interference

Cells (3.5 × 10^5^) were seeded in a six-well plate. After 12 h of incubation, the siRNAs were transfected into cells with siRNA-MateTM transfection reagent (GenePharma, China) for 24 h. Then, the cells were lysed using cell lysis buffer for western blot analysis. The siRNA sequences are shown in Table 1.

**Table 1:** siRNAs used in this study

Primer	Sequence of forward primer	Sequence of reverse primer
G6PD-siRNA-181	GGGUGAUGCCUUCCACCAATT	UUGGUGGAAGGCAUCACCCTT
G6PD-siRNA-241	CCUGGCCAAGAAGAAGAUUTT	AAUCUUCUUCUUGGCCAGGTT
G6PD-siRNA-357	GCAAACAGAGUGAGCCCUUTT	AAGGGCUCACUCUGUUUGCTT
VDAC1-siRNA-168	GUCCGAGAAUGGAUUGGAATT	UUCCAAUCCAUUCUCGGACTT
VDAC1-siRNA-261	CGAGUAUGGGCUGACGUUUTT	AAACGUCAGCCCAUACUCGTT
VDAC1-siRNA-423	GAGGGAGCAUAUCAACCUGTT	CAGGUUGAUAUGCUCCCUCTT
Negative Control	UUCUCCGAACGUGUCACGUTT	ACGUGACACGUUCGGAGAATT

### Immunoprecipitation analysis

For immunoprecipitation, VSMCs were lysed in a buffer previously reported [31] composed of 50 mM Tris–HCl (pH 8.0), 150 mM NaCl, 1.5 mM MgCl_2_, 0.1% sodium dodecyl sulfate (SDS), 0.5% deoxycholate, 0.5% NP-40, 1 mM phenylmethylsulfonyl fluoride (PMSF), and 1 × protease inhibitor cocktail. After centrifugation, the supernatants were first precleared with 30 μL of protein A/G beads (Santa Cruz, sc-2003, USA) to reduce nonspecific binding and immunoprecipitated with 4 μL of specific antibodies for 2 h, followed by incubation with protein A/G-agarose beads at 4 °C overnight. After 12 h of incubation, the immune complexes were centrifuged and washed four times with ice-cold lysis buffer. The immunoprecipitated proteins were further analyzed by Western blotting as described above.

### IP‒LC‒MS/MS analysis

For the IP‒LC‒MS/MS experiment, VSMCs were lysed in IP buffer as described above. One milligram of each sample was IPed with 3 μL of G6PD or IgG antibody as described above. IP’ed proteins were eluted in 5 × loading buffer and separated via SDS‒PAGE. The gel was stained with Coomassie blue, and the gel bands were excised and in-gel digested. Liquid chromatography‒tandem mass spectrometry (LC‒MS/MS) analysis was performed by Jiyun Biotech for the identification of G6PD-interacting proteins.

### Isolation of mitochondria

Mitochondria were extracted using the Minute™ Mitochondria Isolation Kit for Mammalian Cells and Tissues (Invent Biotechnologies, Inc. MP-007, USA). The assay was performed according to the manufacturer’s protocol.

### Glutathione *S*-transferase (GST) pulldown assay

For the interaction of G6PD with VDAC1 in vitro, GST-null and GST-G6PD were produced by *E. coli* BL21 under induction by isopropyl thio-β-d-galactoside (IPTG) at 16 °C. The proteins were purified by affinity absorption using glutathione-Sepharose 4B beads (Amersham Pharmacia Biotech, China). Recombinant GST and GST-G6PD were incubated with total cell lysates of HEK293A cells transfected with Flag-VDAC1 at 4 °C overnight, followed by extensive washing. Proteins on the beads were resolved by 10% SDS-PAGE and visualized by immunoblotting with anti-G6PD and anti-VDAC1 antibodies.

### Plasmid construction and cell transfection

Lipofectamine 2000 (Invitrogen) was used to transfect the plasmids into VSMCs or 293A cells, and siRNA-Mate™ transfection reagent (GenePharma) was used to transfect the siRNAs into VSMCs. All small interfering RNA (siRNA) sequences designed for specific targets are listed in Table 1. We synthesized full-length complementary cDNAs of RAT G6PD and VDAC1 and cloned these cDNAs into the expression vector pcDNA3.1 (Invitrogen) by Syngentech (Beijing, China). G6PD siRNAs, VDAC1 siRNAs, and negative control RNA (si-Ctrl) were designed and synthesized by GeneChem (Shanghai, China).

### Statistical analysis

The experiments were repeated independently at least three times with similar results, and all the results are shown as the mean ± SEM. Data analysis was performed by using GraphPad Prism 9.0 software (GraphPad Software, San Diego, CA, USA) and IBM SPSS Statistics Version 27. For normally distributed data, an unpaired two-tailed Student’s t test, one-way ANOVA, or two-way ANOVA was performed, and *P* values < 0.05 were considered to indicate statistical significance. The representative images were chosen to most accurately represent the group means across the available data, and no data were excluded from the analyses. The investigators were not blinded to the allocation of the animals during the experiments or outcome assessments.

## Results

### G6PD upregulation is correlated with VSMC phenotypic switching

**Fig. 1:**
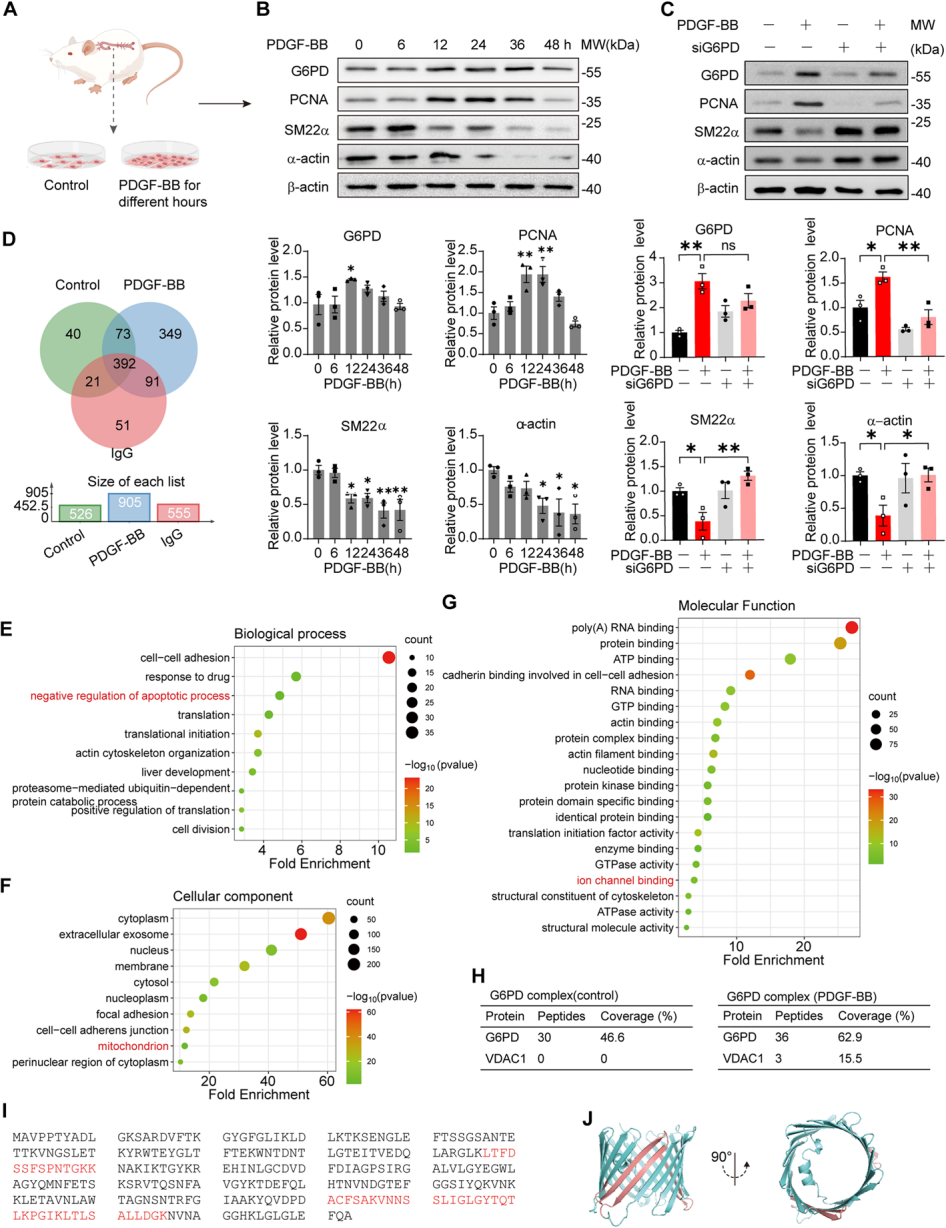
G6PD upregulation promotes VSMC phenotypic switching, as determined by bioinformatics analysis of G6PD-interacting proteins. **A** Schematic diagram of the phenotypic switching of VSMCs induced by platelet-derived growth factor-BB (PDGF-BB) stimulation (20 ng/mL). Primary VSMCs were cultured from the thoracoabdominal aortas of male Sprague–Dawley rats, cultured for 3–5 generations and then stimulated with or without PDGF-BB. The whole-cell lysates were subjected to Western blot analysis. **B** Representative Western blot analysis and analysis of grayscale images of G6PD, PCNA, and VSMC contractile markers (α-actin and SM22α) in the lysates of VSMCs from A, which were treated with PDGF-BB for 0, 12, 24, 36 and 48 h, respectively. β-actin was used as an internal control. The data are presented as the relative fold change at 0 h (*n* = 3). **C** Representative Western blot analysis and analysis of grayscale images of G6PD, PCNA, α-actin, and SM22α in VSMCs stimulated with or without PDGF-BB in response to siG6PD. *n* = 3. **D** Venn diagrams displaying the number of unique and shared proteins identified by LC‒MS between control, PDGF-BB, and IgG VSMCs. **E**–**G** The functional categories based on gene ontology (GO) term enrichment and the signaling pathways based on KEGG enrichment analysis. biological process (**E**), cellular component (**F**), and molecular function (**G**). **H** Mass spectrometry analysis of G6PD and VDAC1 peptides after purification of G6PD-associated proteins. **I**, **J** Three peptides of VDAC1 identified by MS (**I**) and their positions in the 3D structure of VDAC1 (**J**). Statistical significance was determined using one-way ANOVA in (**B**) and two-tailed Student’s *t* tests in (**C**). *P < 0.05; **P < 0.01; ns, no significant difference

To understand the role of G6PD in VSMC phenotypic switching, primary VSMCs from Sprague‒Dawley (SD) rat thoracoabdominal aortic appendages were cultured and stimulated with PDGF-BB (10 ng/mL), a growth factor known to promote VSMC switching in an in vitro model. As presented in Fig. 1A, the cells were cultured for different durations (0–48 h) in serum-free DMEM to gradually induce a proliferative/synthetic phenotype [25]. One of the crucial markers of VSMC phenotypic switching is the loss of SM22α and α-actin, with switched VSMCs displaying a proliferative/synthetic phenotype [32–34]. To test this phenomenon, the protein levels of SM22α, α-actin, PCNA, and G6PD were analyzed at different time points during PDGF-BB stimulation via Western blot analysis. The levels of SM22α and α-actin decreased over time under PDGF-BB stimulation, indicating a gradual switch in VSMCs from a contractile/quiescent phenotype to a synthetic/proliferating phenotype. The level of PCNA, a marker of proliferation, gradually increased at 6 h and peaked at 12 h. Moreover, the expression of G6PD also increased in a time-dependent manner with PDGF-BB stimulation, reaching its peak at 12 h (Fig. 1B). In addition, G6PD is found in diverse oligomeric states, including monomers, dimers, tetramers, and hexamers, but only the dimeric and tetrameric forms are catalytically active [35]. Our observations revealed significant increases in both G6PD dimerization (Additional file 1: Figure S1A) and activity (Additional file 1: Figure S1B) following stimulation with PDGF-BB for 12 h. In summary, G6PD was upregulated and activated in synthetic/proliferated VSMCs, suggesting that G6PD plays an indispensable role in VSMC phenotypic switching.

To determine the essential role of G6PD in PDGF-BB-induced VSMC phenotypic switching, three siRNAs were designed to reduce the expression of G6PD, and siRNA-241 had the greatest interference efficiency (Additional file 1: Figure S1C). Besides, the G6PD activity was tested, and the result showed that knockdown of G6PD reduced the enzyme activity of G6PD in VSMCs (Additional file 1: Figure S1D). Interestingly, the increase in PCNA and decrease in SM22α and α-actin were reversed by siG6PD in proliferative/synthetic VSMCs (Fig. 1C), indicating that G6PD knockdown reversed PDGF-BB-induced VSMC phenotypic switching. To corroborate the findings of G6PD silencing, we studied cultured proliferative/synthetic VSMCs treated with the G6PD inhibitor 6-aminonicotinamide (6AN) [36]. Consistent with the above results, pharmacological inhibition of G6PD also reduced the enzyme activity of G6PD in VSMCs (Additional file 1: Figure S1E). The increase in PCNA and decrease in the contractile markers SM22α and α-actin induced by PDGF-BB were blocked by 6-AN (Additional file 1: Figure S1F). Taken together, these results suggest that increased expression and activity of G6PD contribute to phenotypic switching of VSMCs, which is restrained by pharmacological inhibition or knockdown of G6PD.

### VDAC1 was identified as a novel interacting partner of G6PD

To explore the molecular mechanisms by which G6PD regulates VSMC phenotypic switching, we sought to identify protein complexes that interact with G6PD following stimulation with PDGF-BB. Immunoprecipitation (IP) coupled with mass spectrometry (MS) analysis was performed to systematically analyze G6PD-interacting proteins in VSMCs with (+) or without (−) PDGF-BB stimulation. Rabbit IgG was used as a mock control to exclude nonspecific interactions. The immunoprecipitated proteins were detected using antibodies specific for G6PD or IgG by SDS-polyacrylamide gel electrophoresis and Coomassie Brilliant Blue staining (Additional file 1: Figure S1G).

A total of 526, 905, and 555 proteins were identified in the control, PDGF-BB, and IgG groups, respectively. Venn diagram analysis of the differentially expressed proteins (fold change > 2, *P* < 0.05) between PDGF-BB-treated VSMCs and potential target proteins revealed 360 candidate target proteins, including voltage-dependent anion-selective channel protein 1 (VDAC1) (Fig. 1D). VDAC1 is a multifunctional mitochondrial protein that regulates cellular metabolic and energy functions as well as apoptosis by interacting with a variety of proteins, prompting us to explore its roles in VSMC biology.

A protein‒protein interaction (PPI) regulatory network was constructed with the STRING database and visualized with Cytoscape (Additional file 1: Figure S1H). The CytoNCA plugin was used to calculate the nodes with the highest degree scores [37]. To visualize the data, an online platform (http://www.bioinformatics.com.cn) was used to construct heatmaps and Venn diagrams. Biological process enrichment analysis based on gene ontology (GO) terms was also conducted to explore the possible role of G6PD, which revealed that the proteins associated with the negative regulation of the apoptotic process were significantly enriched (Fig. 1E). Cellular component analysis revealed that the upregulated proteins were enriched in the cytoplasm, extracellular exosome, nucleus, membrane, and mitochondrion (Fig. 1F). The analysis of molecular functions indicated that the upregulated proteins were significantly enriched in poly(A) RNA binding, molecule binding, and ion channel binding (Fig. 1G). Furthermore, to investigate the involvement of development-related signaling pathways, KEGG analysis was conducted, and metabolic pathways were found to be the most prominent component among the enriched pathways (Additional file 1: Figure S1I).

Based on the above findings, VDAC1, a gated protein associated with apoptosis, attracted our attention. The analysis revealed VDAC1 as a major interacting partner of G6PD (Fig. 1H). Three VDAC1 peptides were identified by mass spectrometry (Fig. 1I) and are displayed in the 3D structure of VDAC1 (Fig. 1J), suggesting that G6PD and VDAC1 physically interact and participate in functional crosstalk.

### G6PD translocates to the mitochondria and interacts with VDAC1, influencing mitochondrial function in proliferative VSMCs

**Fig. 2:**
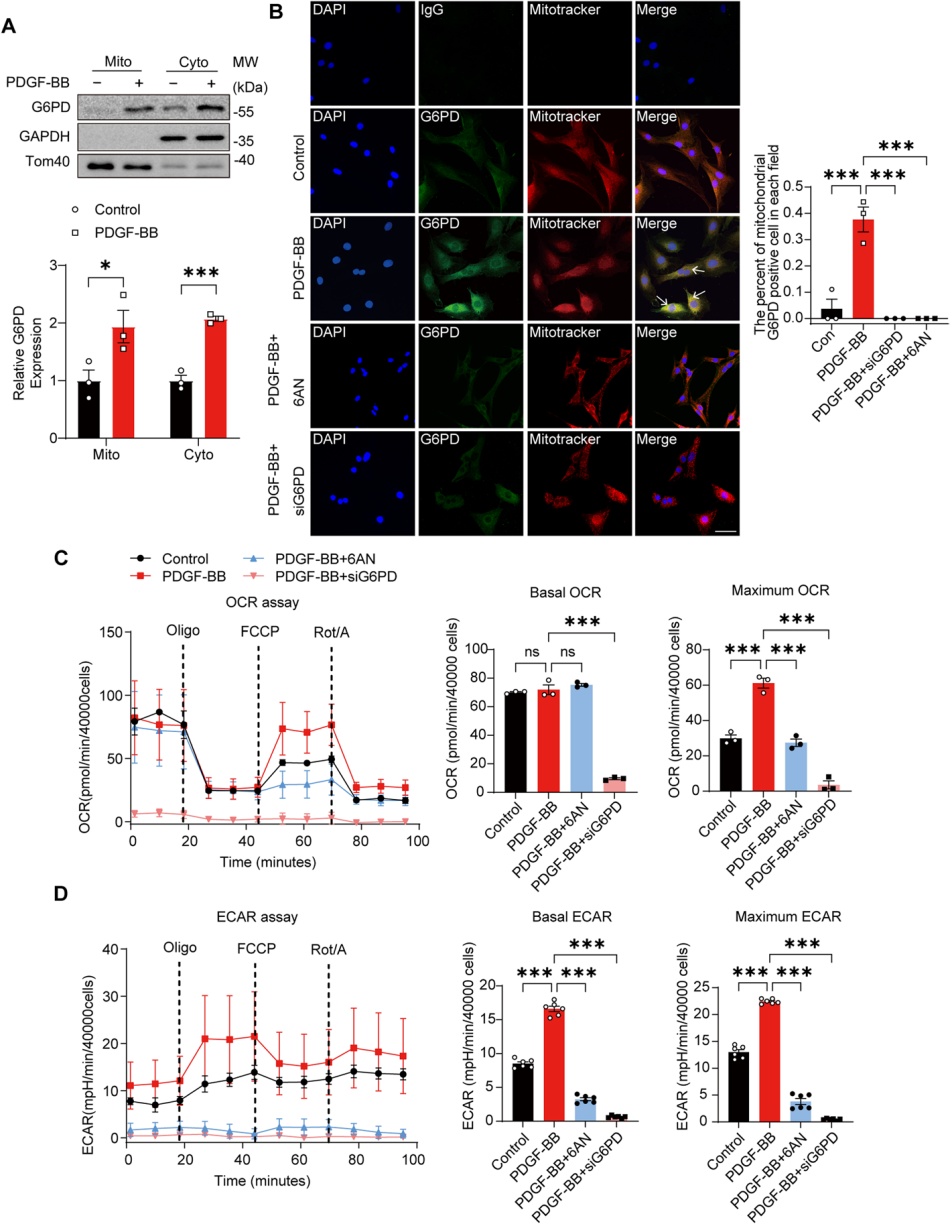
G6PD translocates to mitochondria and affects mitochondrial function. **A** Mitochondrial and cytoplasmic fractions were prepared from VSMCs treated with 20 ng/mL PDGF-BB for 12 h. Western blot analysis of the cytosolic and mitochondrial fractions was performed to evaluate the translocation of G6PD from the cytosolic compartment to the mitochondria. GAPDH and TOM40 were used as the cytosolic and mitochondrial loading controls, respectively. **B** Immunofluorescence analysis was carried out after 12 h of PDGF-BB stimulation, and 6AN and siG6PD were also used in subsequent experiments. Mitochondria were identified with TOM40, nuclei were stained with DAPI, and a G6PD monoclonal antibody was used to indicate endogenous G6PD. Scale bar = 50 µm. *n* = 3. **C**, **D** Seahorse metabolic flux analyses showing the traces and quantification of the basal or maximum mitochondrial oxygen consumption rate (OCR, **C**) and extracellular acidification rate (ECAR, **D**) in VSMCs treated with 6AN and siG6PD under PDGF-BB stimulation. *n* = 3. Statistical significance was determined using two-tailed Student’s *t* tests in (**A**) and one-way ANOVA in (**B**–**D**). *P < 0.05; **P < 0.01; ***P < 0.001

To ascertain the potential interaction between VDAC1, a protein located on the outer mitochondrial membrane [38], and G6PD, we initially evaluated the presence of G6PD in the mitochondria. Proteins extracted from enriched mitochondrial and cytosolic fractions were subjected to Western blot analysis to detect the expression levels of G6PD. The results indicated an increase in G6PD expression in mitochondria following PDGF-BB stimulation (Fig. 2A). To confirm this result, fluorescence colocalization analysis was performed using LAS AF software (Leica Microsystems, Germany). In detail, mitochondria and G6PD were labeled with MitoTracker (red) and a monoclonal anti-G6PD antibody, respectively, followed by a secondary antibody conjugated with FITC (green). As expected, the level of G6PD in mitochondria increased in response to PDGF-BB stimulation (yellow) (Fig. 2B). In addition, we examined the effect of G6PD on mitochondrial function. A Seahorse extracellular flux analyzer was used to measure the oxygen consumption rate (OCR) and extracellular acidification rate (ECAR), which are indirect measures of mitochondrial OXPHOS and glycolysis, respectively. Oligomycin, FCCP (carbonyl cyanide-4-(trifluoromethoxy) phenylhydrazone), and a mixture of rotenone A/antimycin were injected sequentially to assess mitochondrial respiration and nonmitochondrial respiration. The results revealed that the optimal concentration of FCCP for treating VSMCs was 0.5 μM (Additional file 1: Figure S2). Proliferative/synthetic VSMCs displayed a greater maximum OCR and ECAR, suggesting a greater energy demand within these cells. Treatment with 6AN or siG6PD significantly decreased the OCR and ECAR, particularly in G6PD-knockdown cells, in which there was almost a complete decrease in the OCR and ECAR. However, the basal OCR did not significantly differ among the three groups, except for the siG6PD group (Fig. 2C, D). Taken together, these findings suggest that G6PD translocates to mitochondria and influences the function of mitochondria in proliferative/synthetic VSMCs.

**Fig. 3:**
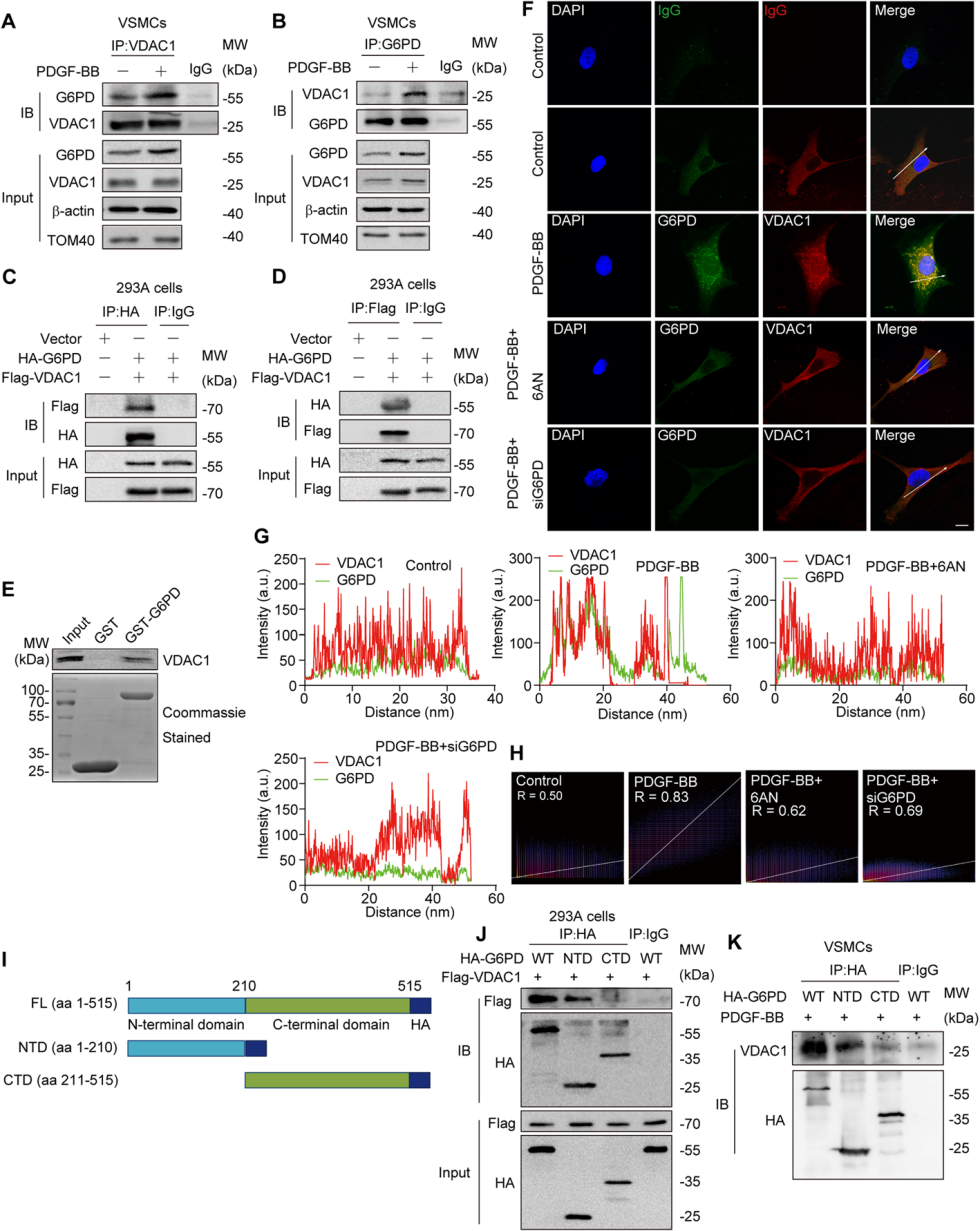
G6PD interacts with VDAC1 under PDGF-BB stimulation. **A**, **B** Endogenous VDAC1–G6PD interactions in VSMCs detected by coimmunoprecipitation (IP) experiments. VSMCs were left unstimulated or stimulated with PDGF-BB for 12 h. Cell lysates were immunoprecipitated (IP) and immunoblotted (IB) with the indicated antibodies. **C**, **D** HEK293A cells were cotransfected with the Flag-VDAC1 plasmid and the HA-G6PD plasmid. The cell lysates were precipitated with an anti-HA or anti-Flag antibody, and the precipitates were analyzed by immunoblotting with an anti-Flag or anti-HA antibody, respectively. **E** GST pulldown analysis using recombinant glutathione S-transferase (GST)-G6PD and Flag-tagged VDAC1 (Flag-VDAC1). **F** The colocalization of G6PD and VDAC1 in the mitochondria of VSMCs stimulated with or without PDGF-BB. Treatment with 6AN and siG6PD was further observed. Scale bar = 10 µm. **G** Histogram showing the arbitrary intensities of G6PD and VDAC1 across the white arrow shown in the zoomed and merged images in **F**. **H** The Pearson correlation coefficient for signal colocalization was determined using Fiji software. **I** Schematic illustration of the G6PD domains used to evaluate the interaction with VDAC1. **J** HEK293A cells were cotransfected with the Flag-VDAC1 plasmid and the HA-G6PD-FL (FL), HA-G6PD-NTD (NTD), or HA-G6PD-CTD (CTD) plasmids. The cell lysates were immunoprecipitated with an anti-HA antibody, and the precipitates were analyzed by immunoblotting with an anti-Flag antibody. **K** VSMCs were transfected with HA-G6PD-FL (FL), HA-G6PD-NTD (NTD), or HA-G6PD-CTD (CTD) plasmids. The cell lysates were immunoprecipitated with an anti-HA antibody, and the precipitates were analyzed by immunoblotting with an anti-VDAC1 antibody

VDAC1 was reported to be expressed in both the mitochondrial and cytoplasmic membranes [39]. To investigate the effect of PDGF-BB stimulation on the localization of VDAC1, we utilized laser confocal microscopy to observe VDAC1 and MitoTracker Red to determine its localization in VSMCs. The results indicated that VDAC1 was localized to mitochondria, regardless of PDGF-BB stimulation (Additional file 1: Figure S3A). In addition, the VDAC1 protein expression underwent a minor change upon PDGF-BB stimulation (Additional file 1: Figure S3B). To further decipher whether VDAC1 is part of the protein complex that interacts with G6PD, coimmunoprecipitation analysis was conducted to assess the interactions between VDAC1 and G6PD. In the VSMC extracts, the G6PD-specific antibody precipitated VDAC1, while the VDAC1-specific antibody precipitated G6PD. Furthermore, the interaction between these two proteins was enhanced after PDGF-BB stimulation (Fig. 3A, B). Notably, the G6PD-specific antibody precipitated VDAC2 and VDAC3, but their interaction was not affected by PDGF-BB stimulation (Additional file 1: Figure S3C, D). In addition, to avoid interference from additional proteins, we synthesized the eukaryotic expression vectors pcDNA3.1-HA-G6PD and pcDNA3.1-Flag-VDAC1 and coexpressed them in 293A cells. Coimmunoprecipitation was subsequently performed using anti-HA or anti-Flag antibodies. Consistent with the findings in VSMCs, the interaction of G6PD with VDAC1 was previously observed (Fig. 3C, D). To determine whether G6PD could directly interact with VDAC1, we performed a GST pull-down assay. Encouragingly, VDAC1 directly interacted with G6PD (Fig. 3E). This hypothesis was further supported by fluorescence colocalization. However, the interaction was reduced when the G6PD concentration was suppressed or pharmacologically inhibited (Fig. 3F–H). To determine the regions within G6PD that are associated with VDAC1, we coexpressed the HA-tagged full length (FL) and truncated N-terminal domain (HA-G6PD-NTD, aa). 1–210), and C-terminal domain (HA-G6PD-CTD, aa. 121–515) of G6PD with Flag-tagged VDAC1 in HEK293T cells (Fig. 3I; Additional file 1: Figure S3E). Specifically, VDAC1 interacted with the wild-type or N-terminal domain but not with the C-terminal domain (Fig. 3J). The G6PD-NTD region was also found to predominantly contribute to this interaction in VSMCs. Collectively, these results provide ample evidence supporting the direct interaction between G6PD and VDAC1, with G6PD-NTD predominantly contributing to this interaction.

### G6PD inhibition induces VSMC apoptosis and decreases cell viability

**Fig. 4:**
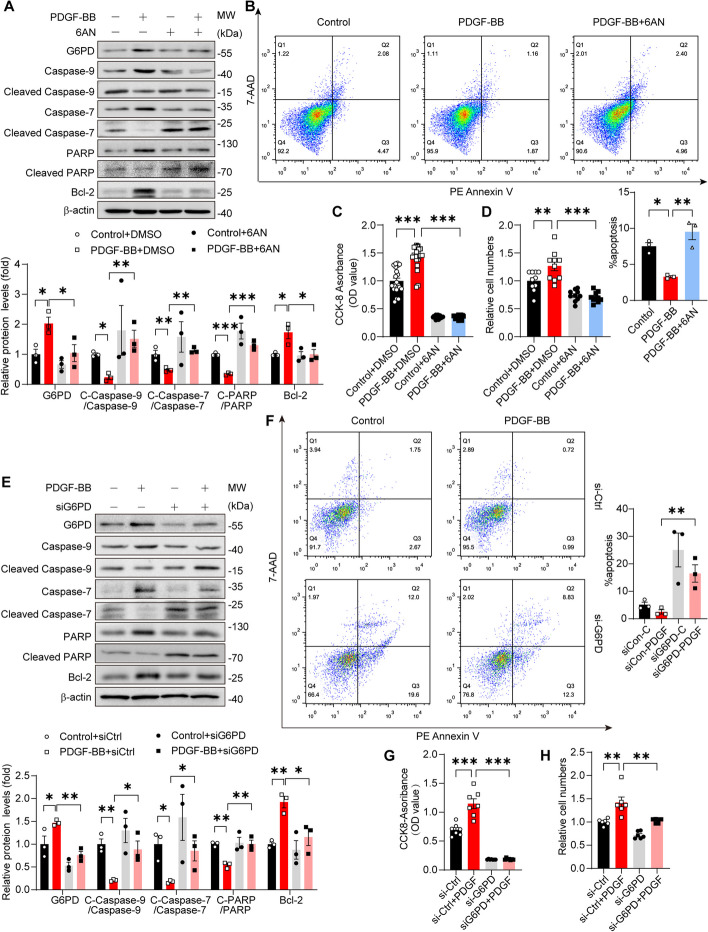
Both pharmacological inhibition and knockdown of G6PD abolish PDGF-BB-induced VSMC apoptosis inhibition and survival. **A** Western blot analysis of caspase-9, cleaved caspase-9, caspase-7, cleaved caspase-7, PARP, cleaved PARP, and Bcl-2 in VSMCs. *n* = 3. **B** Cell apoptosis was evaluated by PE-conjugated Annexin V/7-AAD staining and quantified by flow cytometry. *n* = 3. **C** The viability of VSMCs was assessed by a CCK8 assay. *n* = 22. **D** Cell proliferation was measured by a cell counting experiment. *n* = 10. **E** Western blot analysis of caspase-9, cleaved caspase-9, caspase-7, cleaved caspase-7, PARP, cleaved PARP, and Bcl-2 in VSMCs. *n* = 3. **F** Cell apoptosis was evaluated by PE-conjugated Annexin V/7-AAD staining and quantified by flow cytometry. *n* = 3. **G** The viability of VSMCs was assessed by a CCK8 assay. *n* = 7. **H** Cell proliferation was measured by a cell counting experiment, *n* = 3. Statistical significance was determined using one-way ANOVA. *P < 0.05; **P < 0.01; ***P < 0.001

VDAC1 is a protein channel located in the outer mitochondrial membrane [40, 41]. In addition to its role as an ion channel, VDAC1 is a well-known regulator of cell apoptosis [42–44]. Therefore, our interest was sparked in investigating whether G6PD is involved in mediating apoptosis. We analyzed the expression of various apoptosis-related proteins (Caspase7, Caspase9, PARP, Bcl-2, Cleaved Caspase7, Cleaved Caspase9, and Cleaved PARP) in VSMCs. Synchronously, the levels of the proapoptotic proteins cleaved caspase 7, cleaved caspase 9, and cleaved PARP were decreased, while the level of the antiapoptotic protein Bcl-2 was increased in VSMCs stimulated with PDGF-BB. However, 6AN reversed the changes in the levels of these proteins (Fig. 4A). Apoptosis was detected using the Annexin V FITC Apoptosis Detection Kit from BD Biosciences. Flow cytometry analysis revealed that the apoptosis rate of the untreated VSMCs was approximately 7%. Treatment with PDGF-BB reduced the apoptosis rate of VSMCs to approximately 3.5%, while treatment with 6-AN restored the apoptosis rate to a level similar to that of the control group, which was approximately 9% (Fig. 4B). A Cell Counting Kit-8 (CCK8) assay revealed that VSMC viability was enhanced upon stimulation with PDGF-BB. However, the viability of the VSMCs treated with 6-AN was even lower than that of the control group (Fig. 4C). A Cell Counting Kit-8 (CCK8) assay demonstrated that VSMC viability increased in response to stimulation with PDGF-BB, while the viability of VSMCs treated with 6-AN was lower than that of the control group (Fig. 4D). These findings demonstrated that pharmacologically inhibiting G6PD can enhance VSMC apoptosis and decrease VSMC viability.

To exclude the potential off-target effects of pharmacological inhibitors, siRNA was used to knock down G6PD in proliferative/synthetic VSMCs. Consistent with the pharmacological results, G6PD knockdown increased the levels of the proapoptotic proteins cleaved caspase 7, cleaved caspase 9, and cleaved PARP and reduced the level of the antiapoptotic protein Bcl-2 (Fig. 4E), suggesting that G6PD promoted VSMC apoptosis in the presence or absence of PDGF-BB stimulation, and flow cytometry analysis promoted this conversion (Fig. 4F). Accordingly, CCK-8 and cell counting assays revealed that G6PD silencing suppressed the cell proliferation (Fig. 4G) and viability (Fig. 4H) induced by PDGF-BB. Overall, these findings provide evidence that pharmacological inhibition or silencing of G6PD suppresses VSMC survival by promoting apoptosis and inhibiting proliferation.

### VDAC1 is necessary for G6PD-mediated antiapoptotic effects, and the NTD of G6PD plays a major role in this process

Given that VDAC1 regulates cell apoptosis by interacting with antiapoptotic (Bcl-2 and Bcl-xL) or proapoptotic (Bax, Bak, and Bim) proteins [41], in light of the antiapoptotic function of G6PD in VSMC phenotypic switching and its increased interaction with VDAC1 during the antiapoptotic process, we hypothesized that G6PD could inhibit VSMC apoptosis in a VDAC1-dependent manner. To test our hypothesis, we cotransfected VSMCs with siRNAs targeting VDAC1 and an adenovirus encoding the G6PD open reading frame (Ad-G6PD) and subsequently induced them with PDGF-BB for 12 h. Three siRNAs targeting VDAC1 (si-168, si-261, si-423) were designed, and siRNA-168 had the highest interference efficiency (Additional file 1: Figure S4A). The optimal virus concentrations for cell transfection were 7.5 × 10^7^ PFU/mL for Ad-G6PD and 3.0 × 10^7^ PFU/mL for the control adenovirus (Ad-con) (Additional file 1: Figure S4B).

**Fig. 5:**
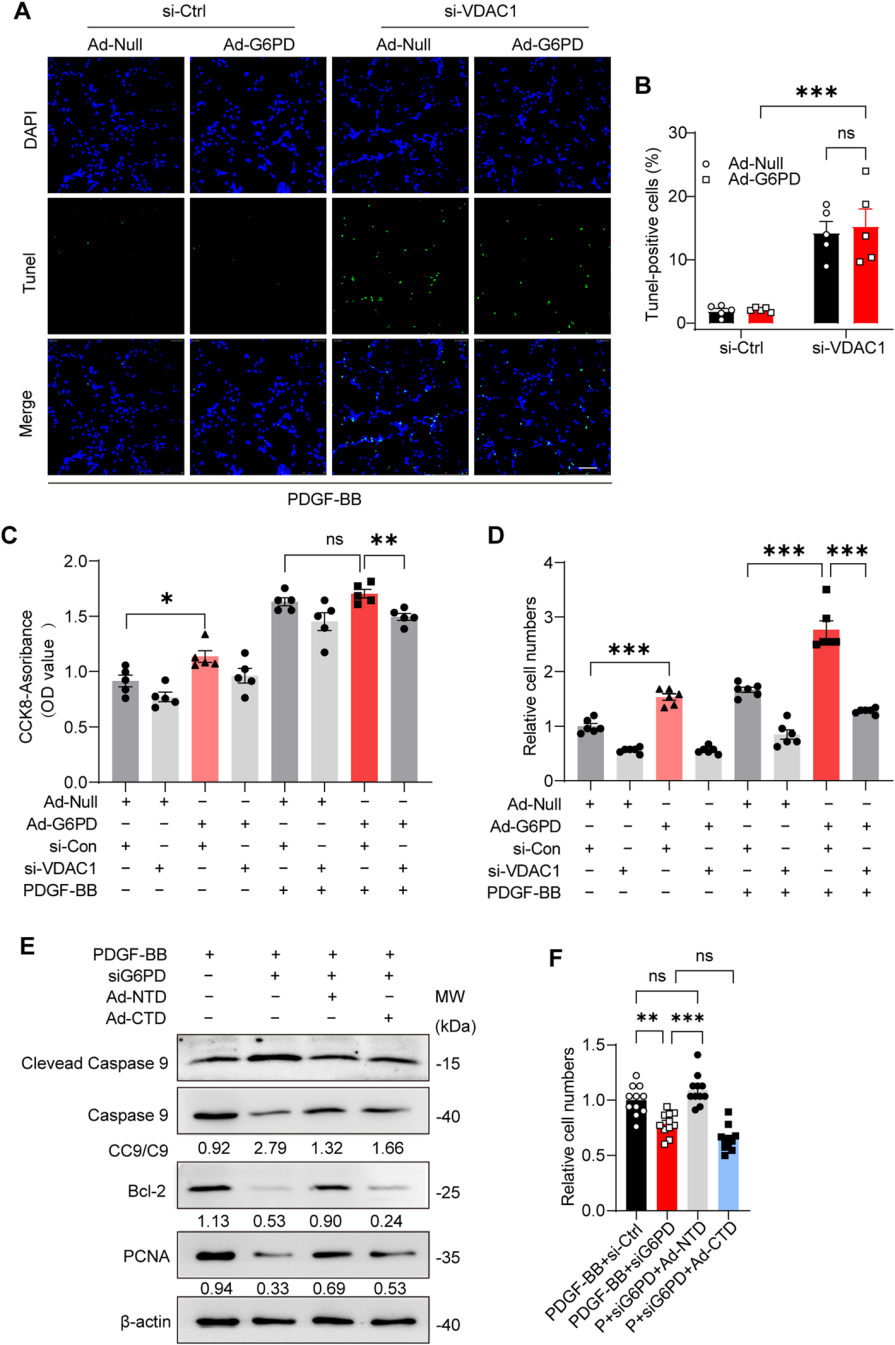
The antiapoptotic effects of G6PD on PDGF-BB-induced VSMC proliferation and viability are mediated by VDAC1. The expression of VDAC1 was reduced by siRNA in VSMCs, and G6PD was subsequently overexpressed by an adenovirus encoding an HA tag. Finally, the cells were treated with PDGF-BB for 12 h. **A**, **B** TUNEL analysis was used to analyze cell apoptosis, and the TUNEL-positive cell ratio was quantified from 5 microscopic views in each experiment. *n* = 5. Scale bar = 100 µm. **C** The viability of VSMCs was assessed by a CCK8 assay. *n* = 5. **D** Cell proliferation was measured by a cell counting experiment. *n* = 6. Ad-Null and si-Con were used as negative controls. **E** G6PD-NTD virus infection inhibited VSMC apoptosis, and western blotting was performed three times; representative images are presented. **F** G6PD-NTD virus infection promoted VSMC survival. Statistical significance was determined using two-way ANOVA in **B**–**D**, **F**. *P < 0.05; **P < 0.01; ***P < 0.001; ns, no significant difference

TUNEL assays showed that silencing VDAC1 led to reduced apoptosis induced by PDGF-BB stimulation, but G6PD overexpression did not reverse this effect (Fig. 5A, B). Knockdown of VDAC1 blocked PDGF-BB-induced cell viability and proliferation, while G6PD overexpression did not reverse these effects, as demonstrated by the CCK-8 and cell counting assays (Fig. 5C, D). Coincidentally, G6PD-NTD was involved mainly in the inhibition of VSMC apoptosis (Additional file 1: Figure S4C; Fig. 5E) and promoted the survival of VSMCs (Fig. 5F). Overall, these findings suggest that VDAC1 is necessary for G6PD-mediated antiapoptotic effects on VSMC phenotypic switching and that the N-terminal domain of G6PD plays a major role in this process.

### G6PD blocks the interaction between VDAC1 and Bax by reducing the oligomerization of VDAC1

**Fig. 6:**
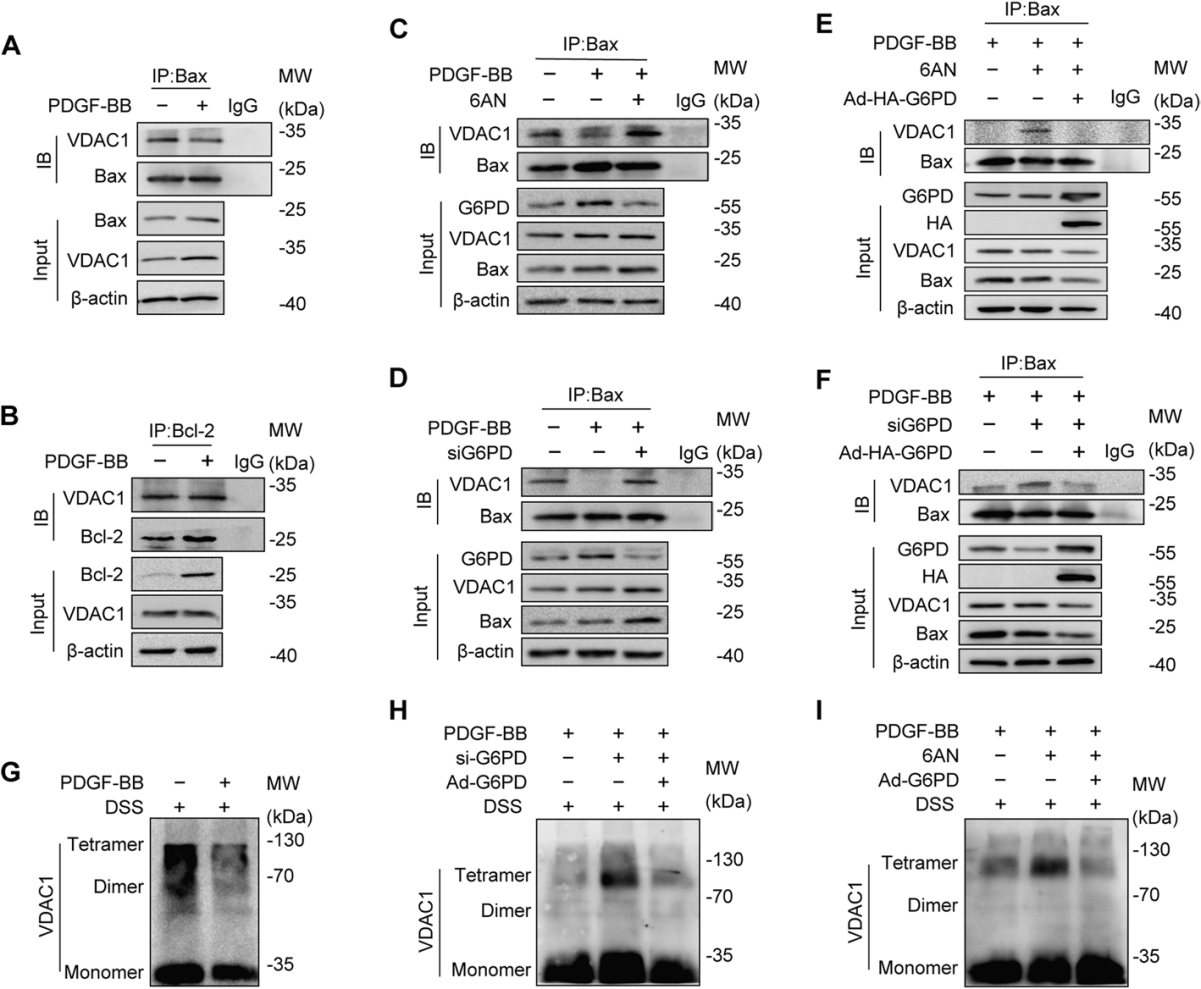
G6PD competes with Bax for binding VDAC1 by reducing its oligomerization. **A**, **B** Coimmunoprecipitation (IP) assays were used to detect the interaction between VDAC1-Bax (**A**) and VDAC1-Bcl-2 (**B**) in VSMCs stimulated with or without PDGF-BB for 12 h, respectively. **C**, **D** The interaction between VDAC1 and Bax in VSMCs was examined by IP after the administration of 6-AN (**C**) or siG6PD (**D**). **E**, **F** After the addition of 6-AN (**E**) or siG6PD (**F**), G6PD was overexpressed, and the interaction between VDAC1 and Bax in VSMCs was examined by IP. **G**–**I** Oligomerization of VDAC1 was detected by WB analysis with or without PDGF-BB stimulation for 12 h (**G**). After treatment with siG6PD (**H**) or 6AN (**I**), G6PD-overexpressing cells were used for further study

Previous studies have demonstrated that VDAC1 can facilitate apoptosis by interacting with the proapoptotic molecule Bax. The molecular mechanism involves Bax translocating from the cytoplasm to the mitochondria and forming a hetero-oligomer with VDAC1 in the outer mitochondrial membrane. This interaction promotes the release of cytochrome c (CytoC) or apoptosis-inducing factor (AIF) from the intermembrane space to the cytoplasm, ultimately leading to apoptosis [45–47]. Furthermore, VDAC1 can also bind to the antiapoptotic protein Bcl-2, thereby inhibiting apoptosis [48]. We investigated whether G6PD is involved in the apoptotic process mediated by Bax or Bcl-2. We employed immunoprecipitation to evaluate the interaction of VDAC1 with Bax or Bcl-2 in the presence or absence of PDGF-BB stimulation. The results indicated that the interaction of VDAC1 with Bax was reduced (Fig. 6A) in PDGF-BB-induced synthetic VSMCs, while the interaction with Bcl-2 remained unchanged (Fig. 6B). Thus, we postulate that G6PD may compete with Bax for binding to VDAC1.

To test our hypothesis, we treated cells with 6-AN or siRNA to inhibit G6PD and subsequently monitored the interaction between VDAC1 and Bax. As anticipated, VDAC1 restored the interaction with Bax when G6PD was knocked down or pharmacologically inhibited (Fig. 6C, D). However, these effects were abolished when G6PD expression was restored via adenovirus (Fig. 6E, F). These results suggest that G6PD suppresses the binding of VDAC1 to Bax, thereby inhibiting VSMC apoptosis.

The process of apoptosis, triggered by the binding of Bax to VDAC1, is often linked to an increase in the oligomerization of VDAC1. On the other hand, a decrease in apoptosis is typically associated with a reduction in VDAC1 oligomerization [49, 50]. To investigate the impact of competitive binding between G6PD and Bax on VDAC1 oligomerization, we first aimed to determine whether VDAC1 oligomerization was reduced in PDGF-BB-induced antiapoptotic VSMCs (Fig. 6G). Pharmacological inhibition or knockdown of G6PD led to the restoration of VDAC1 oligomerization, whereas G6PD overexpression reversed this effect (Fig. 6H, I). Overall, the results suggest that G6PD competes with Bax for binding to VDAC1, leading to a reduction in VDAC1 oligomerization.

### The VDAC1 oligomerization inhibitor VBIT-12 mimics the antiapoptotic function of G6PD

**Fig. 7:**
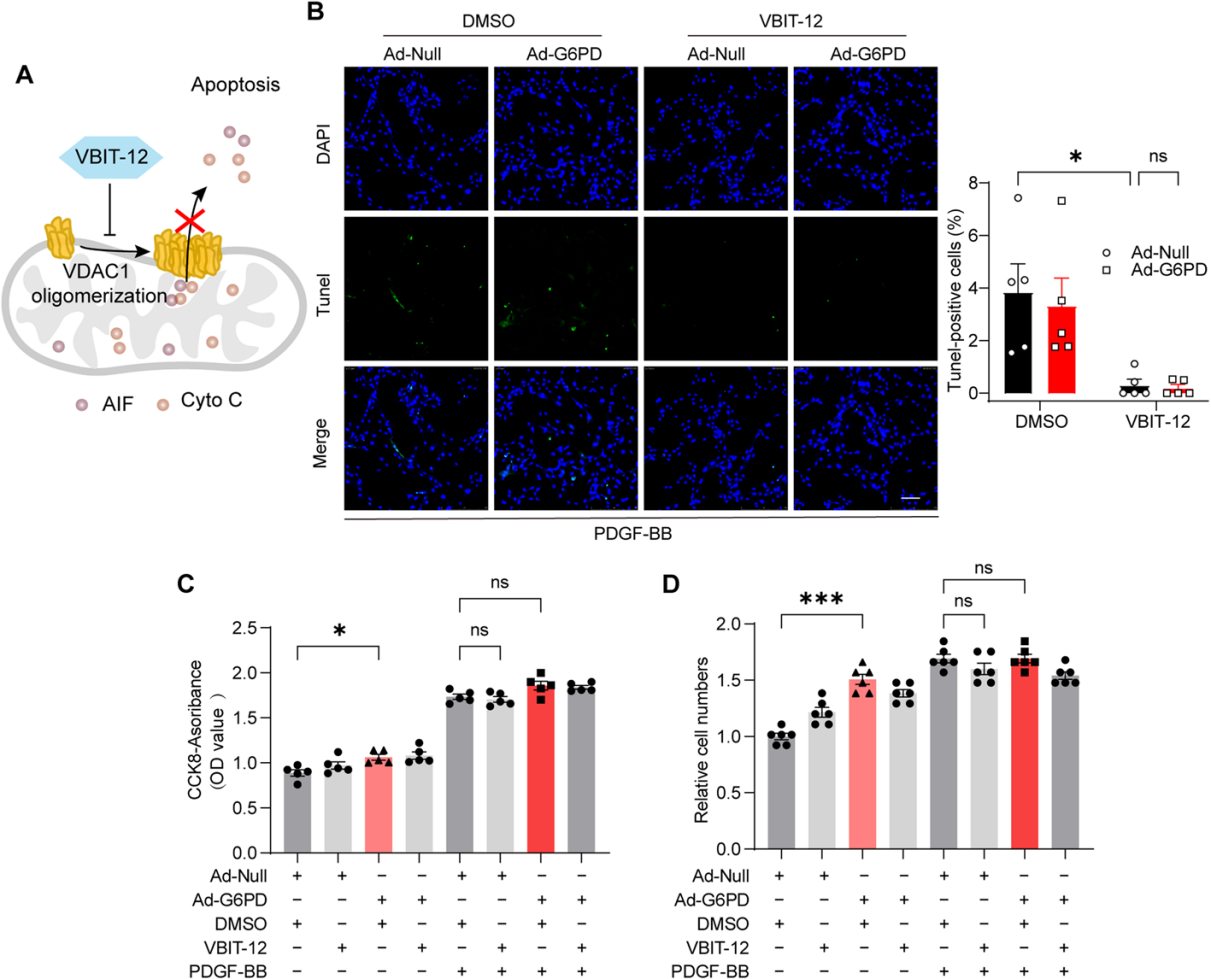
VBIT-12 mimicked the antiapoptotic effect of G6PD. **A** The molecular mechanism by which VBIT-12 inhibits VDAC1 oligomerization and apoptosis resistance. **B** TUNEL analysis was used to analyze cell apoptosis, and the TUNEL-positive cell ratio was quantified in 5 microscopic fields of view in each experiment. Scale bar = 100 µm. **C** The viability of VSMCs was assessed by a CCK8 assay. *n* = 7. **D** Cell proliferation was measured by a cell counting assay. *n* = 6. Ad-Null and DMSO were used as negative controls. Statistical significance was determined using two-tailed Student’s *t* tests (**B**) and two-way ANOVA (**C**, **D**); *P < 0.05; ***P < 0.001; ns, no significant difference

To investigate whether PDGF-BB-induced VSMC apoptosis inhibition was mediated by reduced VDAC1 oligomerization, we treated VSMCs with the VDAC1 oligomerization inhibitor VBIT-12 under PDGF-BB stimulation and then overexpressed Ad-G6PD [38]. VBIT-12 inhibited VDAC1 oligomerization and subsequent apoptosis and associated processes, such as AIF and CytoC release from the mitochondrial intermembrane space (Fig. 7A). Consistent with our hypothesis, similar to G6PD overexpression, VBIT-12 treatment led to a decrease in PDGF-BB-induced VSMC apoptosis, as indicated by the TUNEL assay. However, the effect was only marginally enhanced with additional G6PD overexpression (Fig. 7B). These findings suggest that the inhibition of VDAC1 oligomerization may involve a mechanism similar to that of G6PD-mediated VSMC antiapoptotic effects. Cell viability and proliferation were significantly increased in G6PD-overexpressing VSMCs, but these effects were not evident after further PDGF-BB stimulation. Interestingly, VBIT-12 had no significant effect on cell viability (Fig. 7C) or proliferation (Fig. 7D) mediated by PDGF-BB and G6PD overexpression.

Overall, in synthetic VSMCs, G6PD plays a dual role in promoting cell survival. First, VDAC1 competes with Bax for binding, inhibiting its oligomerization and promoting antiapoptotic cell death. Second, G6PD promotes cell viability and proliferation, thus further promoting VSMC survival.

### G6PD accelerates vascular neointimal hyperplasia by promoting proliferation and inhibiting VSMC apoptosis

**Fig. 8:**
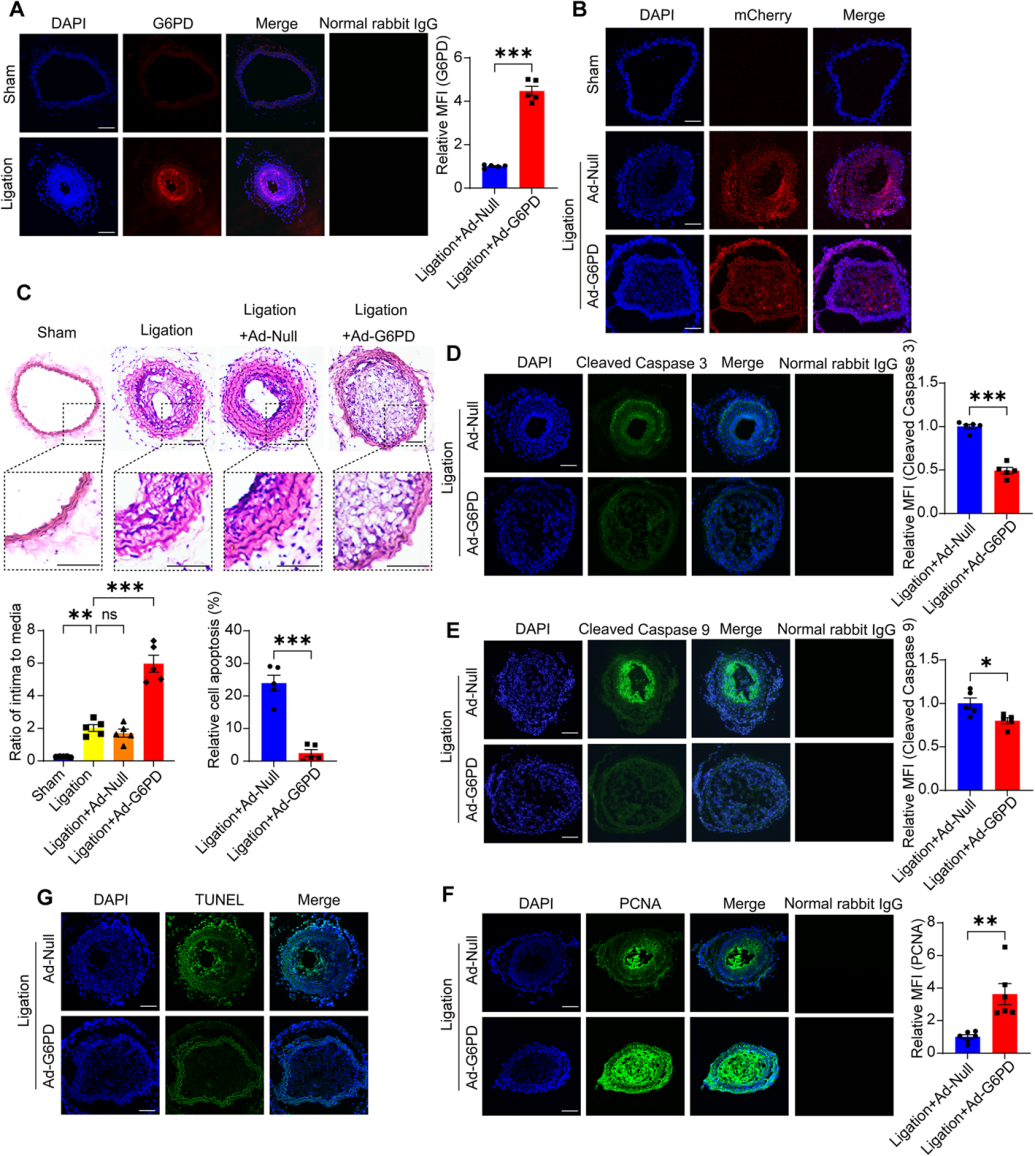
Overexpression of G6PD exacerbates neointimal hyperplasia by decreasing VSMC apoptosis. **A** Representative cross-sections and quantification of G6PD levels following IF staining after ligation surgery of the carotid artery for 14 days. Scale bar = 100 µm. **B** Immunofluorescence of mCherry after 14 days of in situ delivery of Ad-Null or Ad-G6PD into ligated mouse common carotid arteries. Scale bar = 100 µm. **C** Hematoxylin and eosin (HE)-stained cross-sections were obtained, and the intimal/media ratio was measured 14 days after carotid ligation surgery. Scale bar = 100 µm. **D** Immunofluorescence and quantification of cleaved caspase 3 levels after 14 days of in situ delivery of Ad-Null or Ad-G6PD into ligated mouse common carotid arteries. Scale bar = 100 µm. **E** Immunofluorescence and quantification of cleaved caspase 9 levels after 14 days of in situ delivery of Ad-Null or Ad-G6PD into ligated mouse common carotid arteries. Scale bar = 100 µm. **F** Immunofluorescence and quantification of PCNA levels after 14 days of in situ delivery of Ad-Null or Ad-G6PD into ligated mouse common carotid arteries. Scale bar = 100 µm. **G** Immunofluorescence and quantification of TUNEL-positive cells after 14 days of in situ delivery of Ad-Null or Ad-G6PD into ligated mouse common carotid arteries. Scale bar = 100 µm. The data are shown as the mean ± SEM; *n* ≥ 5. Statistical significance was determined using two-tailed Student’s *t* tests (**A**, **D**–**F**) and one-way ANOVA (**C**). *P < 0.05; **P < 0.01; ***P < 0.001; ns, not significant

The in vivo role of G6PD was investigated in a mouse model of neointimal hyperplasia induced by common carotid artery ligation. Two weeks after ligation, the mouse model was established. The expression of G6PD was frequently elevated in the ligated arteries exhibiting neointimal hyperplasia (Fig. 8A).

An adenovirus encoding G6PD bearing a mCherry (red) fluorescence protein (Ad-G6PD) was generated in situ in the ligated arteries, and an adenovirus‐encoding mCherry protein (Ad‐Null) was used as a negative control. Red fluorescence was detected in both arteries and whole blood on day 14, indicating successful in situ delivery (Fig. 8B). After 14 days of in situ delivery, G6PD overexpression exacerbated neointimal formation, as confirmed by an increased intima-to-media ratio (Fig. 8C). Similarly, the levels of the apoptosis-related proteins cleaved caspase 3 (Fig. 8D) and cleaved caspase 9 (Fig. 8E) decreased, while the levels of PCNA (Fig. 8F) and the percentage of TUNEL-positive cells (Fig. 8G) increased. Therefore, G6PD overexpression exacerbates neointimal hyperplasia by promoting proliferation and decreasing VSMC apoptosis.

In addition to increasing apoptosis inhibition and proliferation, the synthetic phenotype also enhances fibrosis and extracellular matrix secretion. IF staining of collagen1 and MMP2 could provide additional evidence that in vivo G6PD overexpression results in vascular dysfunction linked to stenosis or atherosclerosis, due to intimal tunica growth. Our findings demonstrated a notable increase in the extracellular matrix component collagen 1 (Additional file 1: Figure S5A) and the fibrosis marker MMP2 (Additional file 1: Figure S5B) in vascular tissues overexpressing G6PD compared to those in the Ad-Null group, supporting our initial hypothesis.

## Discussion

Herein, we revealed for the first time that G6PD exacerbates neointimal hyperplasia by decreasing VSMC apoptosis. Mechanistically, in synthetic VSMCs induced by PDGF-BB, G6PD interacts directly with the mitochondrial outer membrane channel protein VDAC1. This interaction inhibits Bax-VDAC1 binding-mediated VDAC1 oligomerization and subsequent apoptosis, which is similar to the effect of VBIT-12, a VDAC1 oligomerization inhibitor (graphical abstract). G6PD acts as a novel endogenous connector that links energy metabolism, VSMC phenotypic switching, and neointimal hyperplasia during vascular remodeling. Identifying key factors that promote the VSMC synthesis phenotype and neointimal hyperplasia could lay the foundation for preventing and treating VRDs.

The pathological phenotypic changes that occur in vascular remodeling in cardiovascular disease are primarily attributed to VSMCs [51]. These cells undergo various biological processes such as phenotypic transformation, proliferation, and apoptosis as the disease progresses. Several studies have indicated that G6PD deficiency is associated with an increased risk of cardiovascular disease, suggesting a potential regulatory role of G6PD in VSMCs [52, 53]. VSMCs, located in the middle layer of the vascular wall, can contract and relax, thereby controlling blood flow in the circulatory system. The expression of VSMC contractile phenotype marker genes (*Tagln*, *Myh11*, and *Cnn1*) is crucial for maintaining VSMC differentiation. Conversely, the downregulation of these marker genes can result in VSMC dedifferentiation, ultimately leading to vascular remodeling. Pharmacological inhibition or downregulation of G6PD can promote the expression of VSMC restrictive genes and maintain vascular function [24]. Therefore, G6PD maintains the dedifferentiation state of VSMCs and prevents impairment of vascular function. In addition, G6PD regulates the relaxation and contraction of vascular smooth muscle by changing the opening and closing of ion channels. G6PD can be activated by protein kinase C to induce intracellular free Ca^2+^ to enhance the contraction of VSMCs [26]. In contrast, the inhibition of G6PD relaxes vascular smooth muscle by opening potassium channels [54]. G6PD-mediated metabolites are also involved in the regulation of vascular smooth muscle contraction. NADPH is a metabolite catalyzed by G6PD, which relaxes vascular smooth muscle by inhibiting the formation of the PKG1α dimer [55, 56]. Despite the above studies have demonstrated that G6PD can influence VSMC phenotype by modulating contractile phenotype marker genes, calcium-potassium channels, and metabolites. It is noteworthy that the G6PD identified in this study is distinct and innovative in its correlation with the mitochondrial apoptosis pathway.

Apoptosis plays an important role in VRDs and affects VSMC proliferation, migration, phenotypic switching, lipid metabolism, and inflammation, leading to the formation and development of atherosclerotic plaques and ultimately the progression of atherosclerosis [57, 58]. The occurrence of VSMC apoptosis differs between the early and late stages of atherosclerosis. VSMC phenotypic switching occurs after vascular endothelial damage, where abnormal proliferation and insufficient apoptosis cause VSMCs to migrate from the vascular media to the intima. This leads to the secretion of extracellular matrix and various cytokines, resulting in neointimal hyperplasia [11, 59]. In advanced atherosclerosis, a sustained inflammatory response can cause cell death, which leads to thinning of the fibrous cap and calcification of atherosclerotic plaques. This ultimately results in decreased plaque stability, which can lead to serious clinical complications such as myocardial infarction and stroke [60, 61]. As a result, inhibiting the progression of these pathogenic processes may effectively prevent the development of these diseases. In the intimal hyperplasia stage, VSMCs display resistance to apoptosis. Reducing this resistance has been proven to effectively accelerate VSMC apoptosis and reduce neointimal thickening [62]. Activation of Fas has been shown to increase VSMC apoptosis and reduce neointimal hyperplasia in balloon-injured rat carotid arteries [63]. In this study, we found that the upregulation of G6PD can maintain the VSMC synthetic phenotype by preventing apoptosis. This research could help us gain a better understanding of the pathogenesis of atherosclerosis and provide a foundation for developing more effective prevention and treatment strategies.

For a long time, G6PD was considered to be a protective factor against VRDs. Several studies have shown that individuals with G6PD deficiency exhibit a lower incidence of VRDs than do those without deficiency [64]. However, some studies have reported that individuals with G6PD deficiency have a greater incidence of atherosclerosis and an increased risk of VRDs [52, 65, 66]. Despite both sides presenting a comprehensive argument, the results remain contradictory. Therefore, further research is needed to explore the effect of G6PD on VRD pathogenesis.

Notably, the subcellular localization of G6PD may determine its different functions. G6PD is a recognized cytoplasmic protein that maintains the intracellular redox balance and substance synthesis mainly by regulating the PPP. Our previous study suggested that G6PD binds to SM22α, which promotes its translocation to the cell membrane, increasing NADPH production and aiding in cell redox balance and survival [25]. In hypoxic environments, G6PD may translocate to the nucleus and regulate the expression of genes related to pulmonary hypertension pathogenesis through DNA methylation [67]. In this study, we found that G6PD can translocate to mitochondria and regulate VSMC apoptosis through the intrinsic apoptotic pathway through mitochondria. These findings help to elucidate how G6PD regulates VSMC phenotypic switching and reveal a potential new target for the diagnosis and treatment of VRDs based on VSMC phenotypic switching.

Protein‒protein interactions play a crucial role in regulating biological processes. The combination of liquid chromatography (LC) tandem mass spectrometry (MS/MS) and proteomics through immunoprecipitation (IP) provides a powerful method for studying complex protein‒protein interactions [68]. In our study, we used IP‒LC‒MS/MS analysis to identify a new G6PD-interacting protein, VDAC1. We further confirmed the interaction between the two proteins using co-IP, GST pull-down, and immunofluorescence colocalization. VDAC is the most abundant protein present in the mitochondrial outer membrane. In mammals, three isoforms of VDAC (VDAC1, VDAC2, and VDAC3), which are located on the outer mitochondrial membrane, have been identified. VDAC1 and VDAC2 are known to be associated with cell apoptosis and regulate apoptosis by binding to proapoptotic and antiapoptotic proteins. In contrast, VDAC3 is not typically involved in cell apoptosis [31, 69, 70]. The present study excluded changes in VDAC2 or VDAC3 binding to G6PD before and after PDGF-BB stimulation, as determined via co-IP. A previous study showed that PKM2 is ectopic to mitochondria and binds to VDAC3, which promotes mitochondrial permeability by preventing the ubiquitination and degradation of VDAC3 [31]. Similarly, we observed that G6PD was upregulated in mitochondria and had a greater interaction with VDAC1 in synthetic VSMCs. These findings led us to hypothesize that G6PD may regulate apoptosis by binding to VDAC1. Further functional studies confirmed the antiapoptotic function of G6PD and its dependence on VDAC1.

As apoptosis proceeds, VDAC1 undergoes oligomerization and forms hetero-oligomers with Bax or Bid on the outer mitochondrial membrane. This process leads to the release of cytochrome C and AIF from mitochondria to the cytoplasm, which in turn induces caspase cleavage and promotes the apoptotic process [45–47]. In the present study, we found that the oligomerization of VDAC1 decreased in PDGF-BB-induced antiapoptotic VSMCs, whereas pharmacological inhibition or knockdown of G6PD promoted the oligomerization of VDAC1. This finding suggested a correlation between VDAC1 oligomerization and G6PD expression. Additional co-IP analyses revealed that G6PD and Bax competed for binding to VDAC1, resulting in a decrease in VDAC1 oligomerization and ultimately an antiapoptotic effect. These findings were further validated through the use of VBIT-12, an inhibitor of VDAC1 oligomerization. Through our collective research, we revealed the antiapoptotic mechanism of G6PD in synthetic VSMCs. Specifically, G6PD competes with Bax to decrease VDAC1 oligomerization, which in turn decreases the formation of osmotic pores. This reduction leads to a decrease in the number of apoptotic processes mediated by VDAC1.

Although our study revealed the molecular mechanism by which G6PD regulates VSMC apoptosis and neointimal hyperplasia, we acknowledge the limitations of our study. First, we verified this mechanism at the cellular level. By using VSMC-specific G6PD or VDAC1 knockout mice in vivo to further confirm the function of G6PD in vascular homeostasis, we further verified our conclusions at the overall level, which is important for the prevention and treatment of VRDs. Second, studies have shown that p53 plays a role in inhibiting cancer cell proliferation and promoting apoptosis by inhibiting G6PD [18, 29, 71]. Additionally, p53 can regulate cell apoptosis by binding to VDAC1 [72, 73]. However, it is still unclear whether p53 is involved in G6PD-VDAC1-mediated VSMC apoptosis.

## Conclusions

The present study provides the first evidence that G6PD is involved in resisting VSMC apoptosis through regulating the mitochondrial apoptosis pathway. Specifically, G6PD inhibits VSMC apoptosis by blocking the VDAC1-Bax axis while promoting proliferation and cell viability, thereby promoting VSMC survival, which is conducive to intimal thickening and subsequent VRDs. Targeting the G6PD-VDAC1-Bax axis may contribute to the development of novel therapies for the phenotypic regulation of VSMCs in VRDs. However, further optimization of the potential regulatory mechanisms of G6PD, such as identifying other posttranslational modifications (PTMs) and binding proteins that interact with G6PD, is necessary.

### Supplementary Information

**Additional file 1: Figure S1.** G6PD is upregulated in synthetic VSMCs. **Figure S2.** Changes in the OCR in VSMCs treated with different concentrations of FCCP. **Figure S3.** G6PD translocated to mitochondria and interacted with VDAC1 upon PDGF-BB stimulation. **Figure S4.** Verification of optimal VDAC1 siRNA and virus concentrations. **Figure S5.** Overexpression of G6PD increases fibrosis and extracellular matrix secretion.

**Additional file 2.** Key Resources.

**Additional file 3.** Original western blots.

## Data Availability

The key resources and original western blots are in Additional file 2 and 3, respectively. Other datasets used and analysed during the current study are available from the corresponding author on reasonable request.

## Abbreviations

Bax: Bcl-2-associated X protein
Bcl-2: B-Cell Leukemia/Lymphoma 2
CVD: Cardiovascular disease
G6PD: Glucose-6-phosphate dehydrogenase
GSH: Reduced glutathione
GAPDH: Glyceraldehyde-3-phosphate dehydrogenase
non-oxPPP: Non-oxidative pentose phosphate pathway
oxPPP: Oxidative pentose phosphate pathway
PDGF: Platelet-derived growth factor
PCNA: Proliferating cell nuclear antigen
PPP: Pentose phosphate pathway
R5P: Ribulose-5-phosphate
ROS: Active oxygen
SM22α: Smooth muscle 22 alpha
VSMC: Vascular smooth muscle cell
VDAC: Voltage-dependent anion-selective channel protein
MMP2: Matrix Metalloproteinase 2
