# Supplementary material for: G6PD maintains the VSMC synthetic phenotype and accelerates vascular neointimal hyperplasia by inhibiting the VDAC1–Bax-mediated mitochondrial apoptosis pathway
Source: Cell Mol Biol Lett. 2024 Apr 8;29:47. doi: 10.1186/s11658-024-00566-w (PMC11003121; doi:10.1186/s11658-024-00566-w)

**Additional Figures**


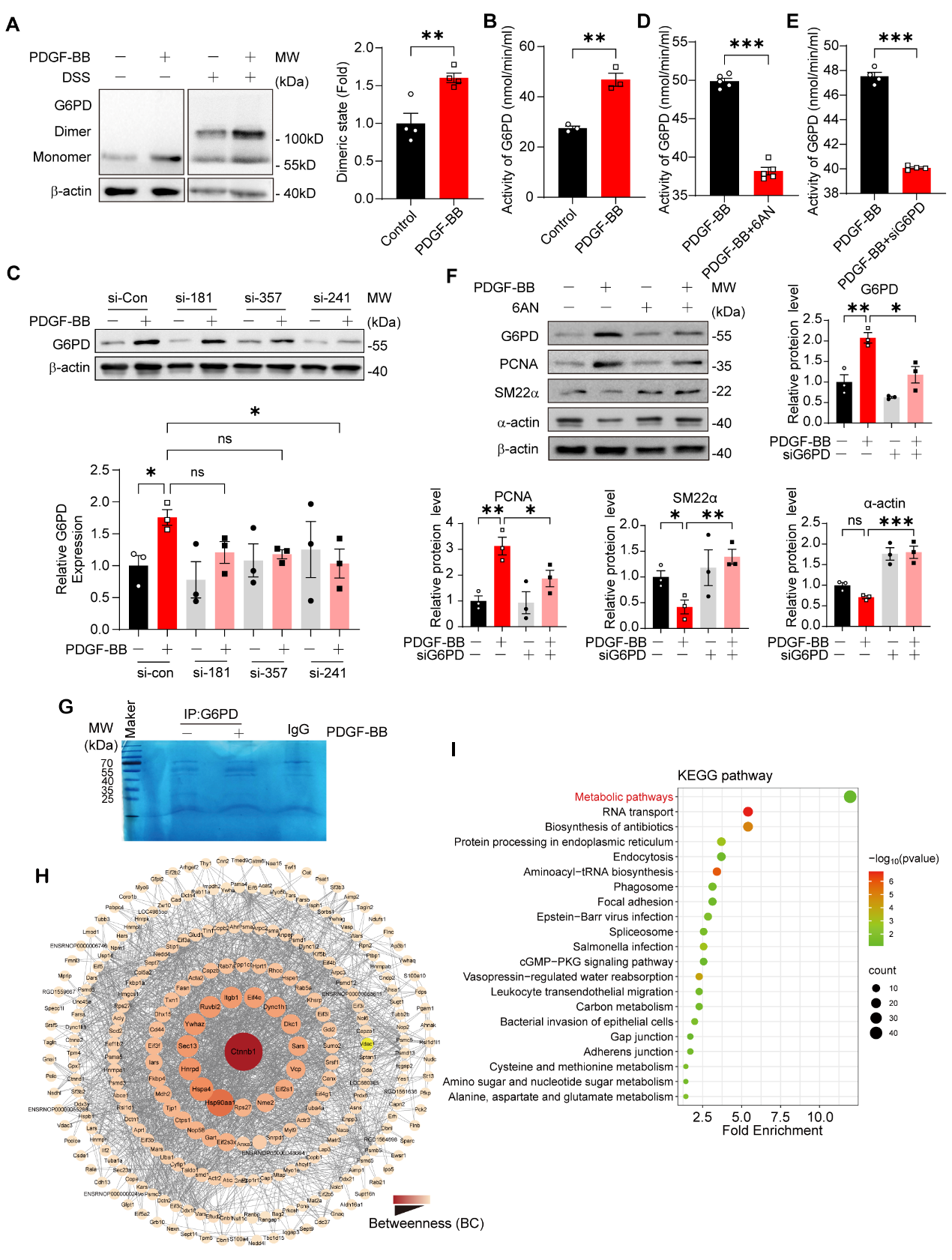


**Fig. S1 G6PD is upregulated in synthetic VSMCs.**

(A) Analysis of oligomeric states of G6PD by the chemical cross-linking agent DSS. (B) G6PD enzymatic activity was measured in VSMCs with or without PDGF-BB stimulation. n=3. (C) Validation of G6PD‐specific siRNAs. The knockdown efficiency of each G6PD-specific siRNA was detected by Western blot analysis using the corresponding specific antibodies. (D) G6PD enzymatic activity was measured in VSMCs in response to siG6PD or not with PDGF-BB stimulation. n=5. (E) G6PD enzymatic activity was measured in VSMCs in response to 6AN or not with PDGF-BB stimulation. n=5. (F) Representative Western blot analysis and analysis of the grayscale image of G6PD, PCNA, α-actin, and SM22α in VSMCs in response to 6AN or not with PDGF-BB stimulation. n = 3. (G) Coomassie-stained SDS‒PAGE gel of G6pd-bound proteins. After PDGF-BB stimulation of VSMCs, IP experiments were performed using a G6PD monoclonal antibody in whole-cell lysates. SDS‒PAGE separation was then performed, followed by LC-MS analysis to identify new interacting proteins of G6PD. The image of Coomassie's brilliant blue staining for the gel is shown. (H) A PPI network of the putative G6PD binding partners was built according to the STRING database. The size of the nodes corresponds to their BC values. BC, betweenness centrality. (I) The signaling pathway is based on KEGG enrichment analysis. Statistical significance was determined using two-tailed Student’s t-test in (A, B, D and E) and one-way ANOVA in (C and F), **P* < 0.05; ***P* < 0.01; ****P* < 0.001; ns, no significant difference.


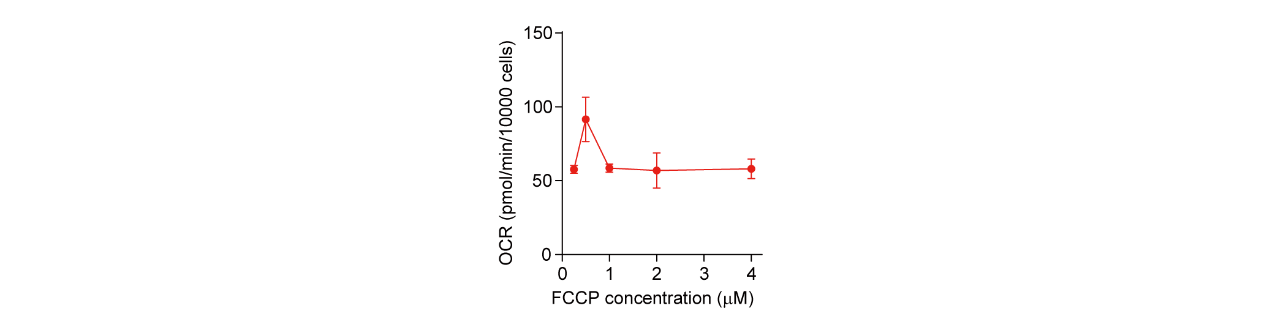


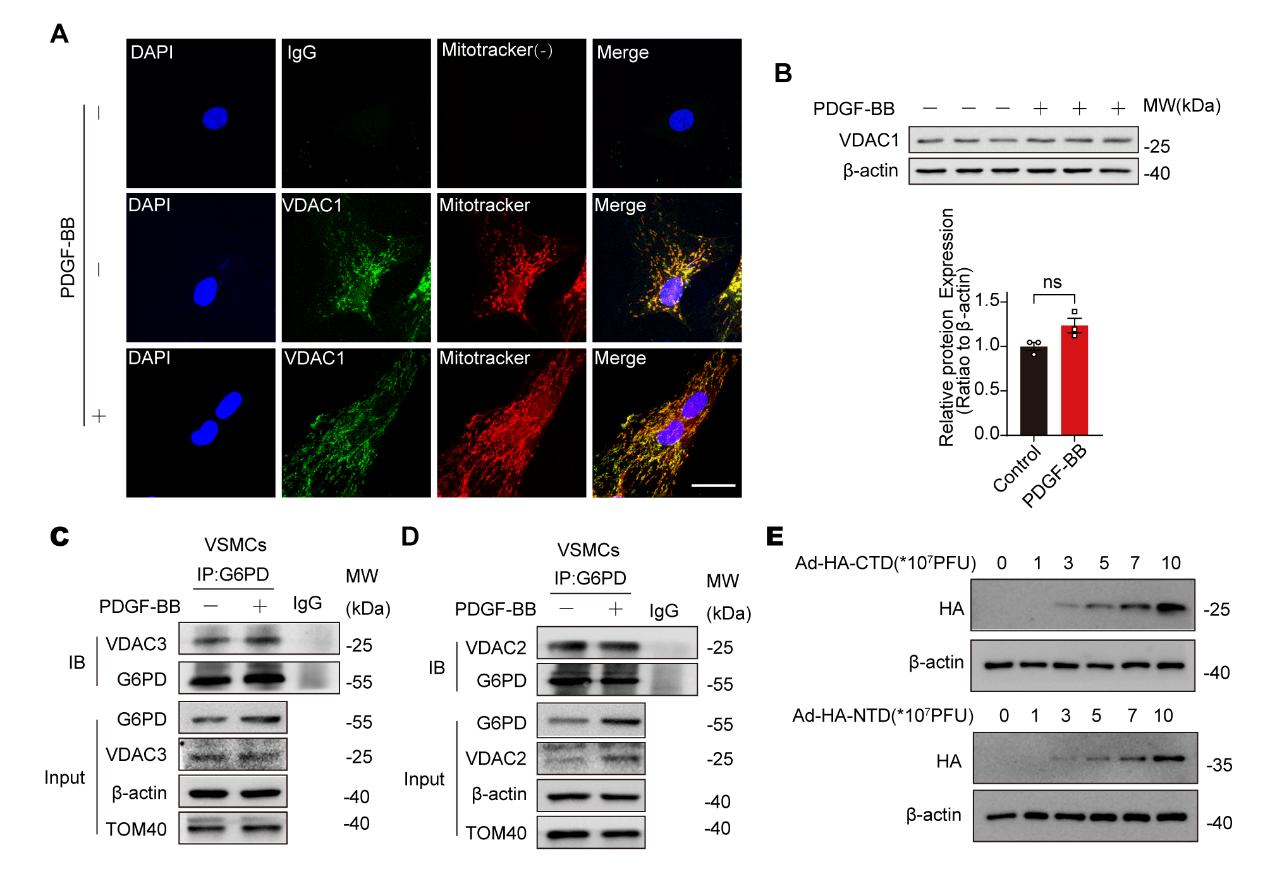
**Fig. S2 Changes in the OCR in VSMCs treated with different concentrations of FCCP. n = 6.**

**Fig. S3 G6PD translocated to mitochondria and interacted with VDAC1 upon PDGF-BB stimulation.**

(A) Immunofluorescence staining for endogenous VDAC1 and mitochondria in VSMCs stimulated with or without PDGF-BB. Scale bar = 25 µm. (B) Western blotting analysis and quantification of the protein levels of VDAC1 in cells upon PDGF-BB stimulation or not under static conditions for 12 h. n = 3. (C) Endogenous VDAC2–G6PD interactions in VSMCs detected by coimmunoprecipitation (IP) experiments. (D) Endogenous VDAC3–G6PD interactions in VSMCs detected by coimmunoprecipitation (IP) experiments. Statistical significance was determined using two-tailed Student’s t-tests in (B). (E) The optimal transfection concentrations of Ad-HA-NTD and Ad-HA-CTD were detected by Western blotting. ns, no significant difference.


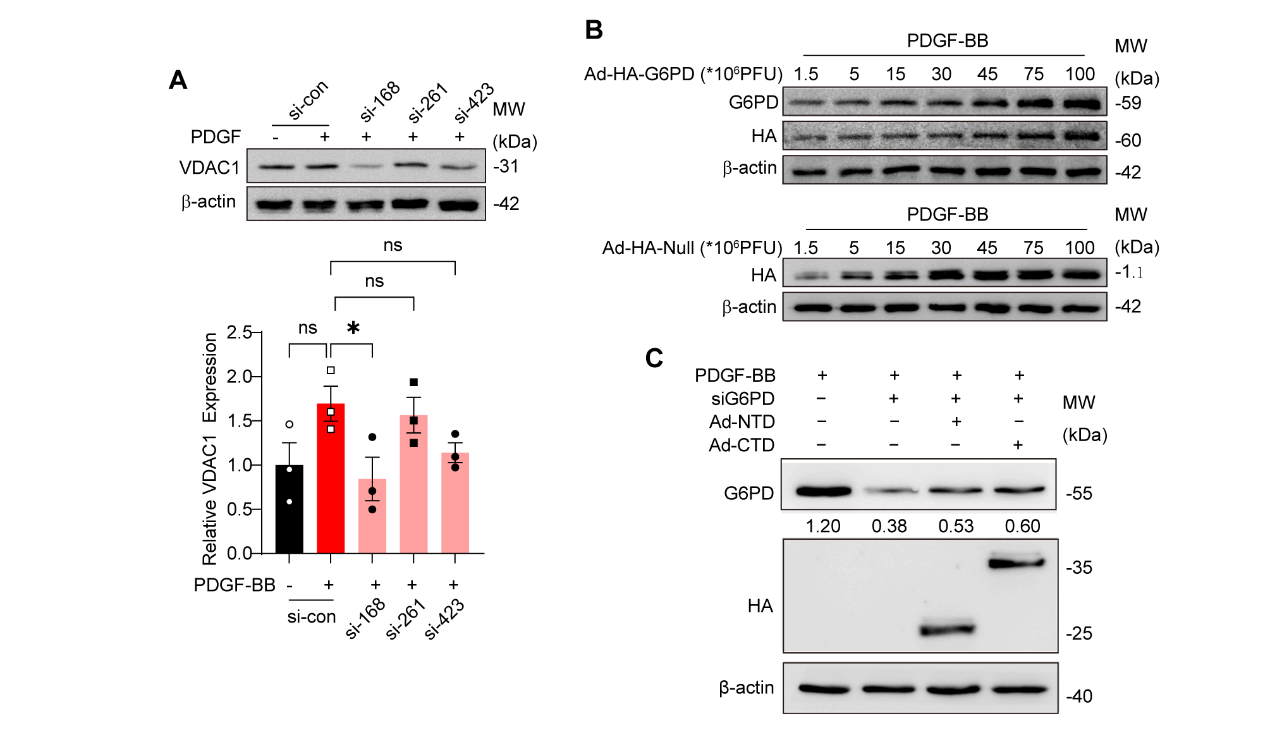


**Fig. S4 Verification of optimal VDAC1 siRNA and virus concentrations.**

(A) Knockdown efficiency of VDAC1‐specific siRNAs was detected by western blotting (WB) using a VDAC1-specific antibody. (B) The optimal transfection concentrations of Ad-HA-G6PD and Ad-Null were detected by Western blotting. (C) The G6PD level and the transfection of Ad-HA-NTD and Ad-HA-CTD were detected by Western blotting. Statistical significance was determined using one-way ANOVA in A, **P* < 0.05; ns, no significant difference.


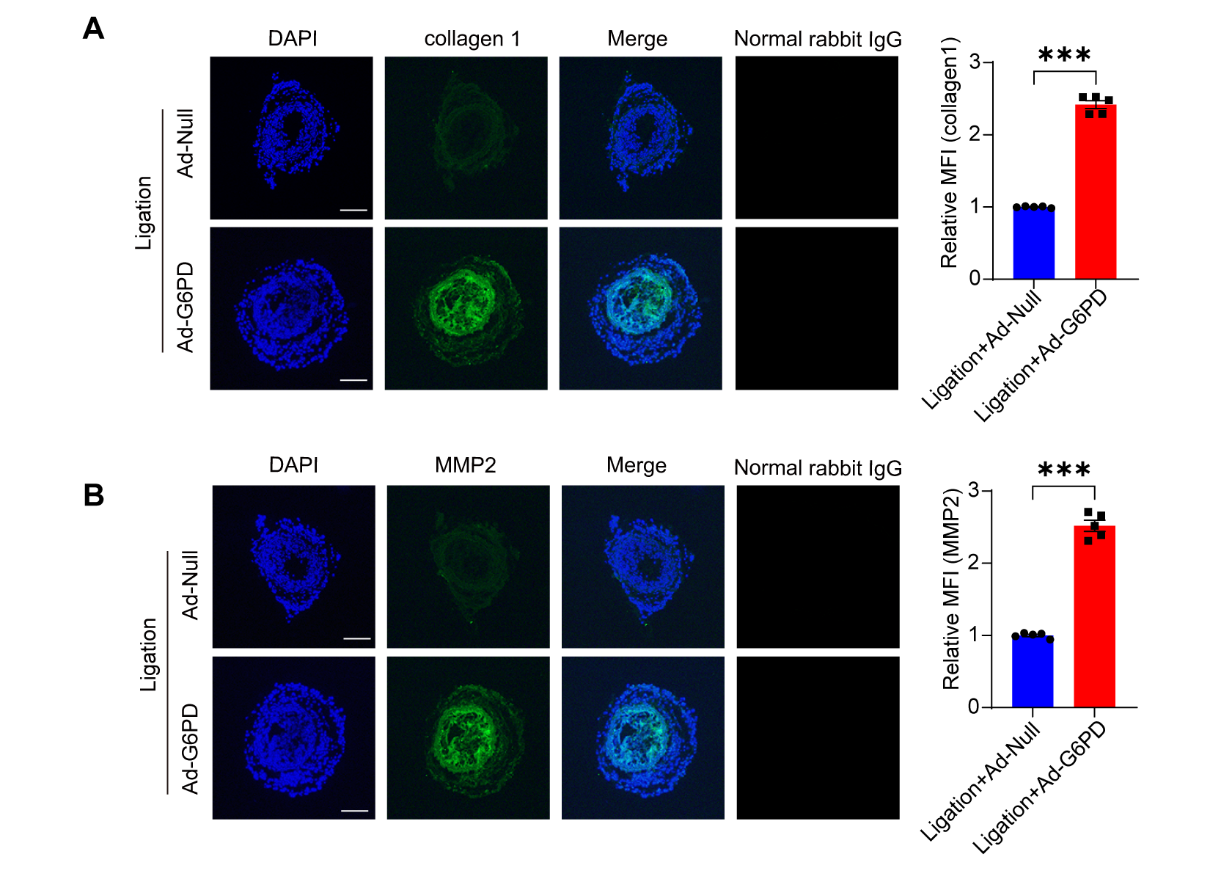
 **Fig. S5 Overexpression of G6PD increases fibrosis and extracellular matrix secretion.**

(A) Immunofluorescence and quantification of collagen1 levels after 14 days of in situ delivery of Ad-Null or Ad-G6PD into ligated mouse common carotid arteries. Scale bar=100 µm.

(B) Immunofluorescence and quantification of MMP2 levels after 14 days of in situ delivery of Ad-Null or Ad-G6PD into ligated mouse common carotid arteries. Scale bar=100 µm.

The data are shown as the mean ± SEM; n ≥ 5. Statistical significance was determined using two-tailed Student’s t tests (A and B). ****P* < 0.001.
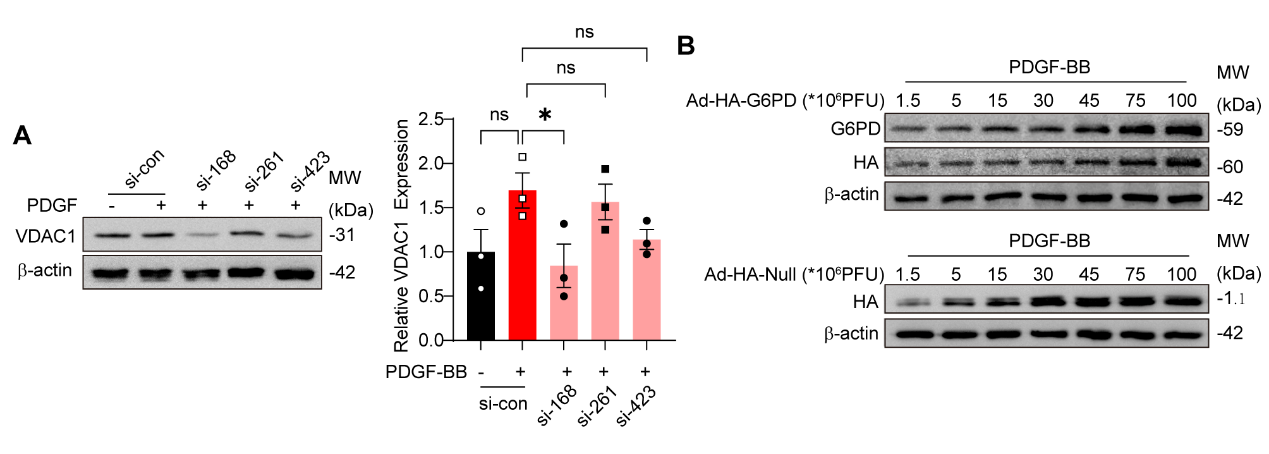

Supplement: Supplementary file 1 — Additional file 1: Figure S1. G6PD is upregulated in synthetic VSMCs. Figure S2. Changes in the OCR in VSMCs treated with different concentrations of FCCP. Figure S3. G6PD translocated to mitochondria and interacted with VDAC1 upon PDGF-BB stimulation. Figure S4. Verification of optimal VDAC1 siRNA and virus concentrations. Figure S5. Overexpression of G6PD increases fibrosis and extracellular matrix secretion. [file 11658_2024_566_MOESM1_ESM.docx]
