# Supplementary material for: G6PD maintains the VSMC synthetic phenotype and accelerates vascular neointimal hyperplasia by inhibiting the VDAC1–Bax-mediated mitochondrial apoptosis pathway
Source: Cell Mol Biol Lett. 2024 Apr 8;29:47. doi: 10.1186/s11658-024-00566-w (PMC11003121; doi:10.1186/s11658-024-00566-w)
Supplement: Supplementary file 2 — Additional file 2. Key Resources. [file 11658_2024_566_MOESM2_ESM.docx]

| **Key Resources Table** | | | | |
| --- | --- | --- | --- | --- |
| **Reagent type (species) or resource** | **Designation** | **Source or reference** | **Identifiers** | **Additional information** |
| Antibody | Rabbit monoclonal anti- G6PD | Abcam | Cat# ab210702; RRID: AB_2923527 | Dilution (1:1000) |
| Antibody | Rabbit monoclonal anti-VDAC1 | Abcam | Cat# ab14734;  RRID: AB_443084 | Dilution (1:1000) |
| Antibody | Rabbit monoclonal anti-VDAC1 | PTM Biosciences | Cat# PTM-6157 | Dilution (1:1000) |
| Antibody | Rabbit monoclonal anti-Bax | Abways | Cat# CY5059 | Dilution (1:2000) |
| Antibody | Rabbit monoclonal anti-Bcl-2 | Abways | Cat# CY6717 | Dilution (1:500) |
| Antibody | Rabbit polyclonal anti-SM22α | Abcam | Cat# ab14106;  RRID: AB_443021 | Dilution (1:1000) |
| Antibody | Mouse monoclonal anti-PCNA | PTM bio | Cat# PTM-5187 | Dilution (1:1000) |
| Antibody | Rabbit polyclonal anti-VDAC2 | Wanlei bio | Cat# WL05474 | Dilution (1:500) |
| Antibody | Mouse monoclonal anti-VDAC3 | Proteintech | Cat# 55260-1-AP;  RRID:AB_10973676 | Dilution (1:500) |
| Antibody | Rabbit monoclonal anti-β-actin | PTM bio | Cat# PTM-5436 | Dilution (1:1000) |
| Antibody | Rabbit monoclonal anti-α-actin | PTM bio | Cat# PTM-6373 | Dilution (1:1000) |
| Antibody | Mouse monoclonal anti-GAPDH | Proteintech | Cat# 60004-1-Ig; RRID: AB_2107436 | Dilution (1:1000) |
| Antibody | Rabbit polyclonal anti-Tom40 | ABclonal | Cat# A3213;  RRID: AB_2764992 | Dilution (1:1000) |
| Antibody | Mouse monoclonal anti-Caspase 9 | PTM bio | Cat# PTM-5311 | Dilution (1:1000) |
| Antibody | Rabbit polyclonal anti-Cleaved Caspase 9 | Wanlei bio | Cat# WL01838 | Dilution (1:1000) |
| Antibody | Rabbit monoclonal anti-Caspase 7 | PTM bio | Cat# PTM-5297 | Dilution (1:500) |
| Antibody | Rabbit polyclonal anti- Caspase 7/Cleaved Caspase 7 | Wanlei bio | Cat# WL02360 | Dilution (1:500) |
| Antibody | Rabbit polyclonal anti-Caspase 3/Cleaved Caspase 3 | Wanlei bio | Cat# WL02117; RRID: AB_2910623 | Dilution (1:500) |
| Antibody | Mouse monoclonal anti-DDDDK(GST) | MBL | Cat# M185-3L;  RRID:AB 11123930 | Dilution (1:10000) |
| Antibody | Mouse monoclonal anti-HA | MBL | Cat# M180-3;  RRID:AB_10951811 | Dilution (1:10000) |
| Antibody | Rabbit polyclonal anti-PARP/Cleaved PARP | Wanlei bio | Cat# WL01932;  RRID: AB_2910624 | Dilution (1:500) |
| Antibody | HRP mouse anti-rabbit IgG | Proteintech | Cat# SA00001-2  RRID: AB_2722564 | - |
| Antibody | HRP goat anti-mouse IgG | Proteintech | Cat# SA00001-1  RRID: AB_2722565 | Dilution (1:1000) |
| Antibody | CoraLite594-conjugated Recombinant Rabbit Anti-Mouse IgG Kappa Light Chain | Proteintech | Cat# SA00014-2  RRID: AB_2935612 | Dilution (1:50) |
| Antibody | CoraLite488-conjugated Goat Anti-Rabbit IgG(H+L) | Proteintech | Cat# SA00013-2  RRID: AB_2797132 | Dilution (1:50) |
| Bacterial | DH5α competent E. coli | Biomed | Cat# EC0112 | - |
| Bacterial | BL21 competent E. coli | Biomed | Cat# BC201-01 | - |
| Chemical compound, drug | VBIT-12 | Selleck | Cat# S8936 | in DMSO |
| Chemical compound, drug | 6-Aminonicotinamide (6AN) | [MCE](https://www.medchemexpress.cn/6-aminonicotinamide.html) | Cat# HY-W010342 | in DMSO |
| Chemical compound, drug | DMSO | Sigma | Cat# D2650 | -- |
| Chemical compound, drug | Tween 20 | Fisher Bioreagents | Cat# BP 337-500 | - |
| Chemical compound, drug | RIPA buffer | Sigma | Cat# R0278 | - |
| Chemical compound, drug | FBS | Gibco | Cat# 10099141C | - |
| Chemical compound, drug | Agarose | Invitrogen | Cat# R0491 | - |
| Chemical compound, drug | Recombinant Rat PDGF-BB Protein | R&D | Cat# 520-BB-050/CF | - |
| Chemical compound, drug | penicillin-streptomycin | Sigma | Cat# TMS-AB2 | - |
| Chemical compound, drug | DAPI | Invitrogen | Cat# D1306 | - |
| Chemical compound, drug | PMSF | Sigma | Cat# 329-98-6 | - |
| Chemical compound, drug | DMEM low glucose | Gibco | Cat# 11885092 | - |
| Chemical compound, drug | pancreatin | Amresco | Cat# 8049-47-6 | - |
| Chemical compound, drug | Paraformaldehyde,4％ | Solarbio | Cat# P1110 | - |
| Chemical compound, drug | Western Blotting Luminol Reagent | Santa cruz | Cat# sc-2048 | - |
| Chemical compound, drug | PVDF Membrane | Milipore | Cat# IPVH00005 | - |
| Chemical compound, drug | MitoTracker™ Red CMXRos Dye | Invitrogen | Cat# M46752 | Mitochodria specific fluorescent dye |
| Chemical compound, drug | GST-tag Purification Resin | Beyotime | Cat# P2250 | - |
| Chemical compound, drug | Lipofectamine 2000 | Invitrogen | Cat# 11668019 | - |
| Chemical compound, drug | siRNA-Mate^TM^ transfection reagent | Gene Pharma | Cat# G04009 | - |
| Chemical compound, drug | Triton (x100) | Sigma-aldrich | Cat# 1086431000 | - |
| Chemical compound, drug | BCA Protein Assay Kit | Pierce^TM^ Thermo Scientific | Cat# 23225 | - |
| Chemical compound, drug | Trypan blue (0,4%) | Gibco | Cat# 15250-061 | - |
| Chemical compound, drug | Ethylenediaminetetraacetic acid (EDTA) | Thermo scientific | Cat# J15694-AP | - |
| Chemical compound, drug | DMEM, high glucose | Gibco | Cat# 11965118 | - |
| Chemical compound, drug | Disuccinimidyl suberate (DSS) | Sigma-Aldrich | Cat# S1885 | - |
| Chemical compound, drug | Penicillin and streptomycin | Sigma-Aldrich | Cat# P4333 | - |
| Chemical compound, drug | PBS, 1× | Absin | Cat# abs962 | - |
| Commercial assay or kit | One Step TUNEL Apoptosis Assay Kit | Beyotime Biotechnology | Cat# C1088 | - |
| Commercial assay or kit | Seahorse XF Cell Mito Stress Test Kit | Agilent | Cat# 103015-100 | - |
| Commercial assay or kit | G6PD Activity Assay Kit | Cayman | Cat# 700300 | - |
| Commercial assay or kit | Minute^TM^ Mitochondria Isolation Kit for Mammalian Cells and Tissues | Invent Biotechnologies | Cat# MP-007 | - |
| Commercial assay or kit | Cell Counting Kit-8 | Beyotime Biotechnology | Cat# C0037 | - |
| Commercial assay or kit | PE Annexin V Apoptosis Detection Kit I | BD Biosciences | Cat# 559763 | - |
| Cell line (H. sapiens) | HEK293A | This paper | N/A | - |
| Sequence-based reagent | G6PD-F | This paper | PCR primers | CGGAATTCATGGCAGAGCAGGTGGCTT |
| Sequence-based reagent | G6PD-R | This paper | PCR primers | CCCTCGAGTCAGAGCTTGTGAGGGTTC |
| Sequence-based reagent | G6PD-siRNA-181-F | This paper | PCR primers | GGGUGAUGCCUUCCACCAATT |
| Sequence-based reagent | G6PD-siRNA-181-R | This paper | PCR primers | UUGGUGGAAGGCAUCACCCTT |
| Sequence-based reagent | G6PD-siRNA-241-F | This paper | PCR primers | CCUGGCCAAGAAGAAGAUUTT |
| Sequence-based reagent | G6PD-siRNA-241-R | This paper | PCR primers | AAUCUUCUUCUUGGCCAGGTT |
| Sequence-based reagent | G6PD-siRNA-357-F | This paper | PCR primers | GCAAACAGAGUGAGCCCUUTT |
| Sequence-based reagent | G6PD-siRNA-357-R | This paper | PCR primers | AAGGGCUCACUCUGUUUGCTT |
| Sequence-based reagent | VDAC1-siRNA-168-F | This paper | PCR primers | GUCCGAGAAUGGAUUGGAATT |
| Sequence-based reagent | VDAC1-siRNA-168-R | This paper | PCR primers | UUCCAAUCCAUUCUCGGACTT |
| Sequence-based reagent | VDAC1-siRNA-261-F | This paper | PCR primers | CGAGUAUGGGCUGACGUUUTT |
| Sequence-based reagent | VDAC1-siRNA-261-R | This paper | PCR primers | AAACGUCAGCCCAUACUCGTT |
| Sequence-based reagent | VDAC1-siRNA-423-F | This paper | PCR primers | GAGGGAGCAUAUCAACCUGTT |
| Sequence-based reagent | VDAC1-siRNA-423-R | This paper | PCR primers | CAGGUUGAUAUGCUCCCUCTT |
| Sequence-based reagent | Negative Control-F | This paper | PCR primers | UUCUCCGAACGUGUCACGUTT |
| Sequence-based reagent | Negative Control-R | This paper | PCR primers | ACGUGACACGUUCGGAGAATT |
| Recombinant DNA reagent | HA-G6PD | This paper | N/A | - |
| Recombinant DNA reagent | Flag-VDAC1 | This paper | N/A | - |
| Recombinant DNA reagent | GST-G6PD | This paper | N/A | - |
| Software, algorithm | Fiji/ImageJ | ImageJ | https://imagej.nih.gov/ij/ | - |
| Software, algorithm | Graphpad’s Prism 9.00 software | GraphPad | https://www.graphpad.com/ | - |
| Software, algorithm | FlowJo | TreeStar | N/A | - |
