# Supplementary material for: G6PD maintains the VSMC synthetic phenotype and accelerates vascular neointimal hyperplasia by inhibiting the VDAC1–Bax-mediated mitochondrial apoptosis pathway
Source: Cell Mol Biol Lett. 2024 Apr 8;29:47. doi: 10.1186/s11658-024-00566-w (PMC11003121; doi:10.1186/s11658-024-00566-w)

Uncropped gels

Fig .1B

G6PD

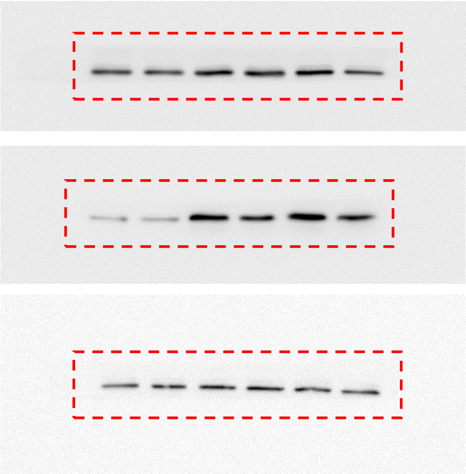

SM22α

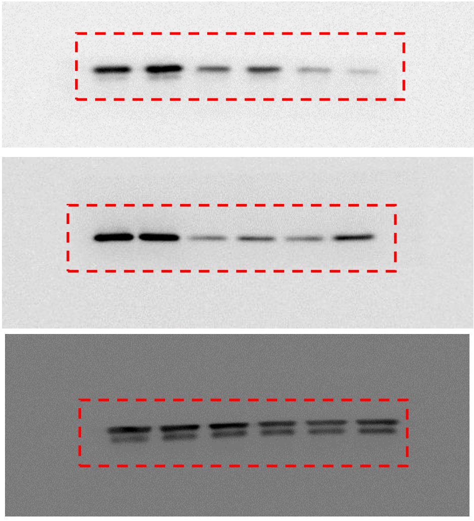

PCNA

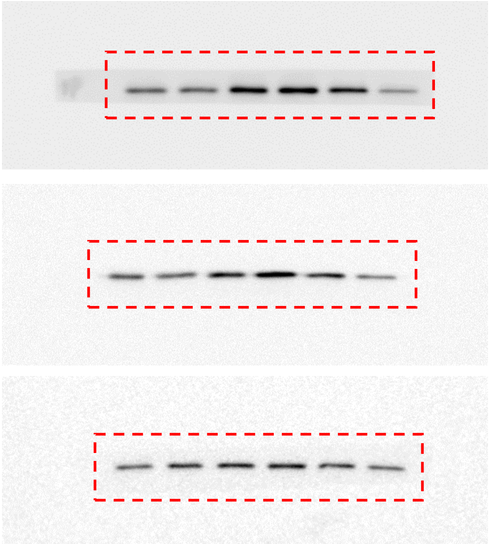

β-actin

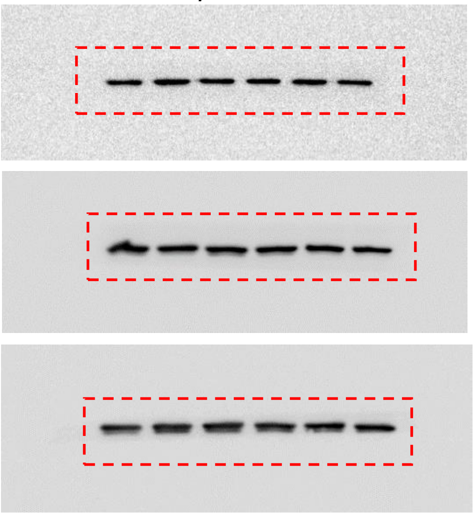

α-actin

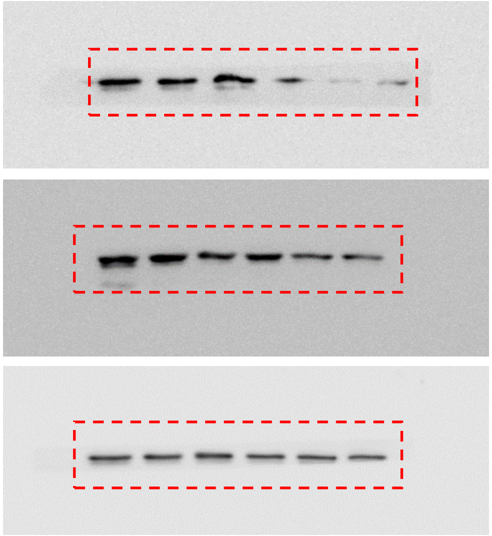

Fig .1C

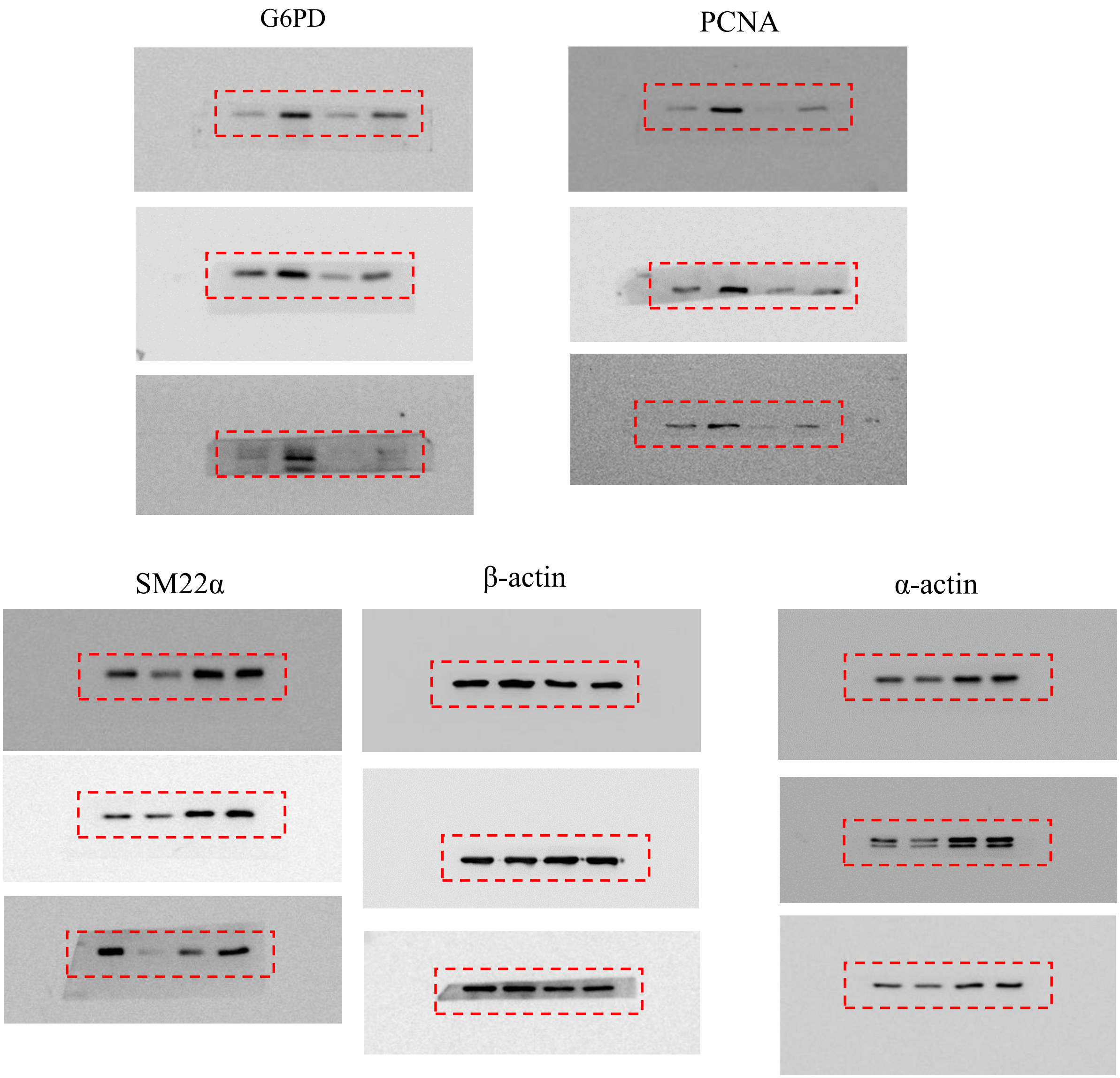

Fig .2A

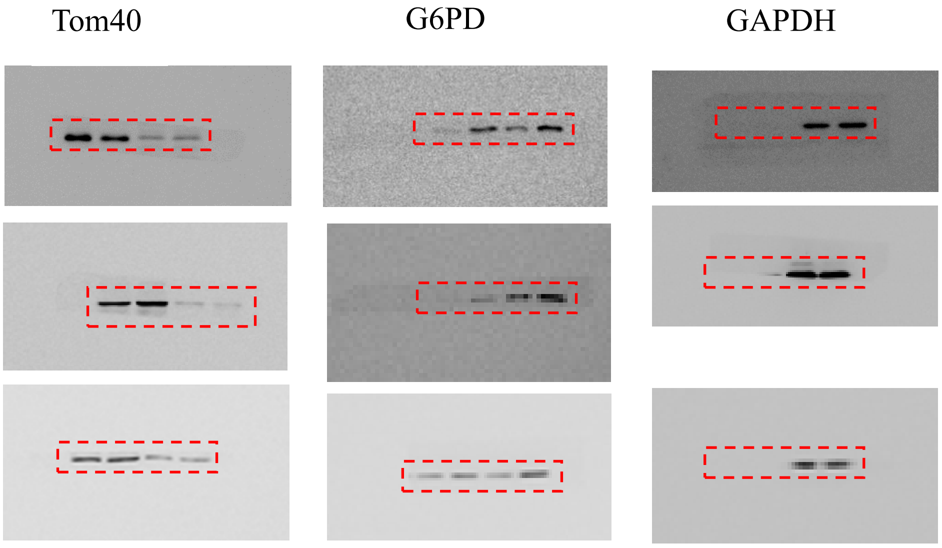

Fig .3A

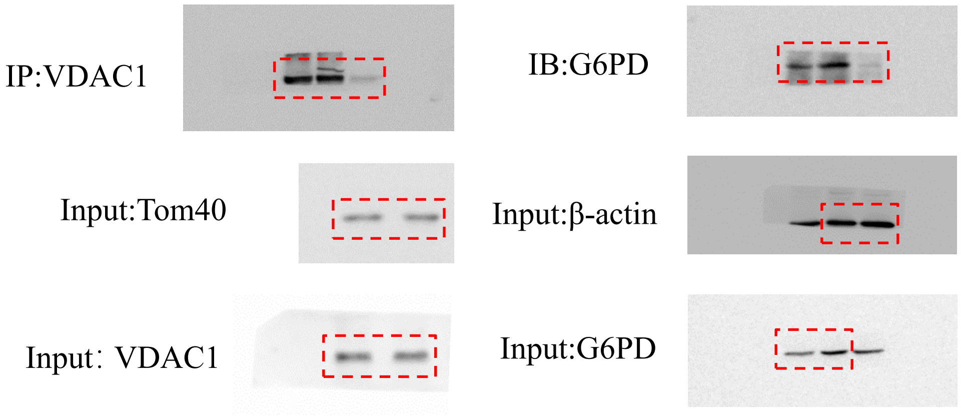

Fig .3B

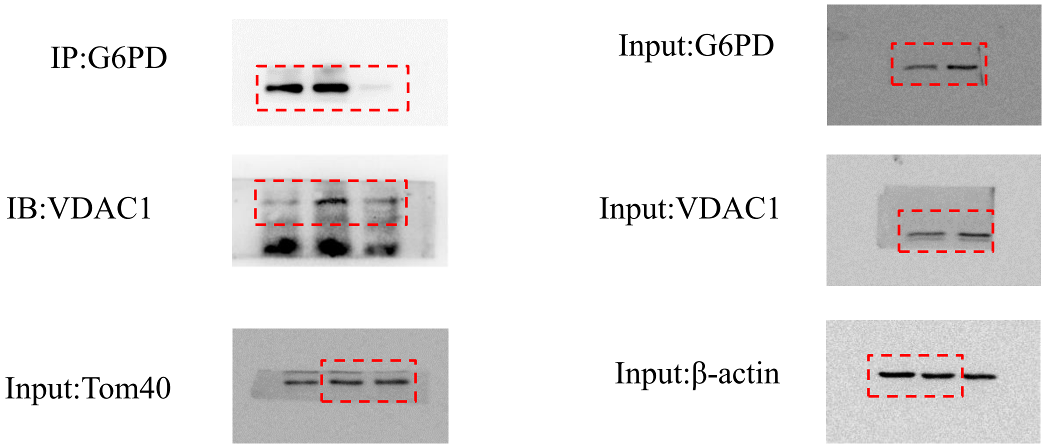

Fig .3C

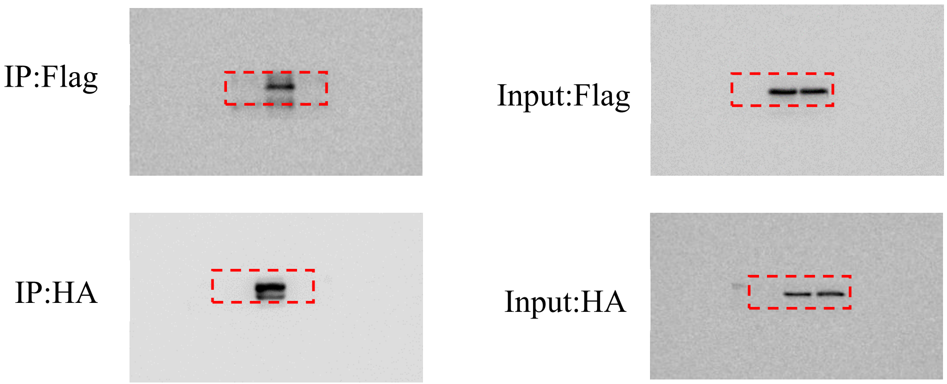

Fig .3D

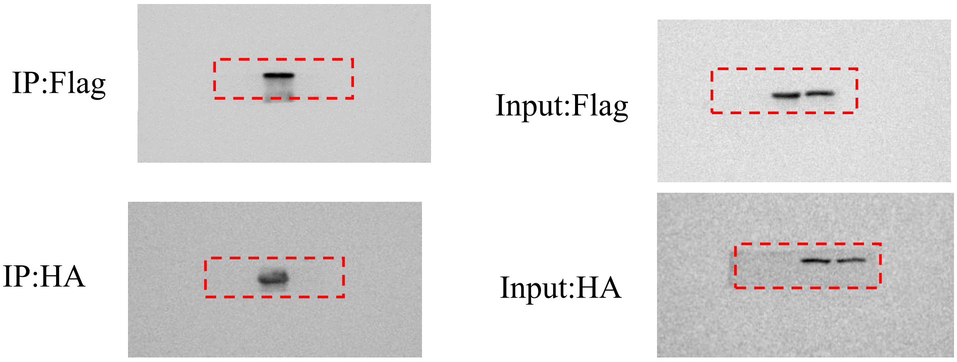

Fig .3E

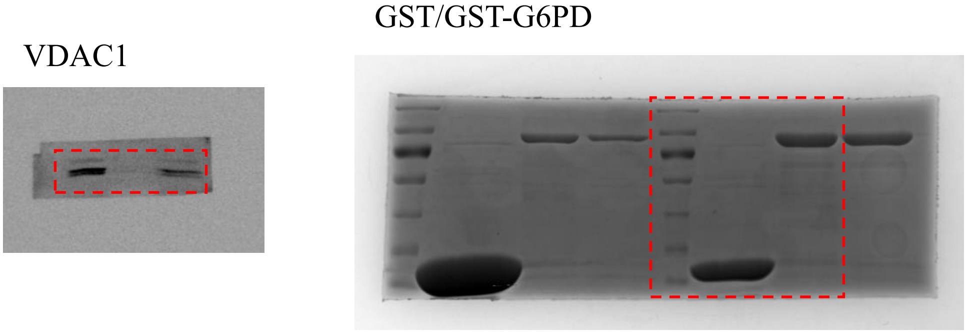

Fig .3J

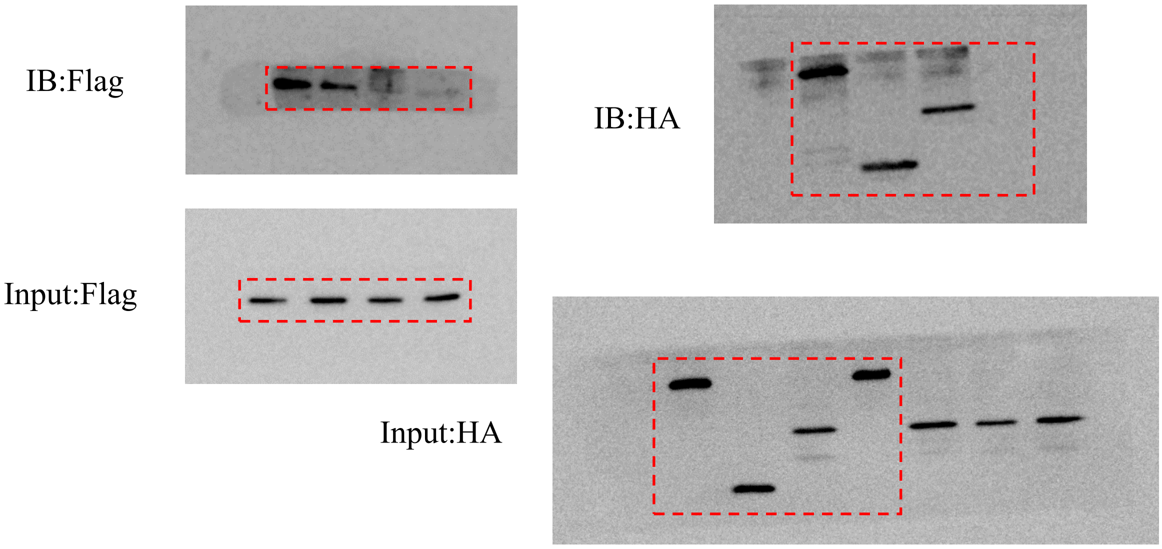

Fig.3K

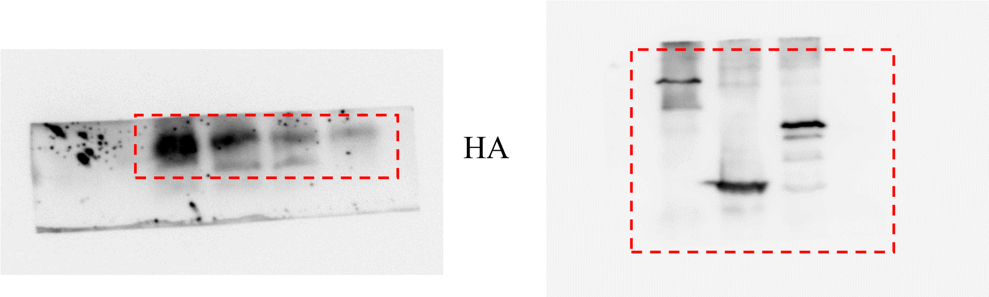

Fig .4A

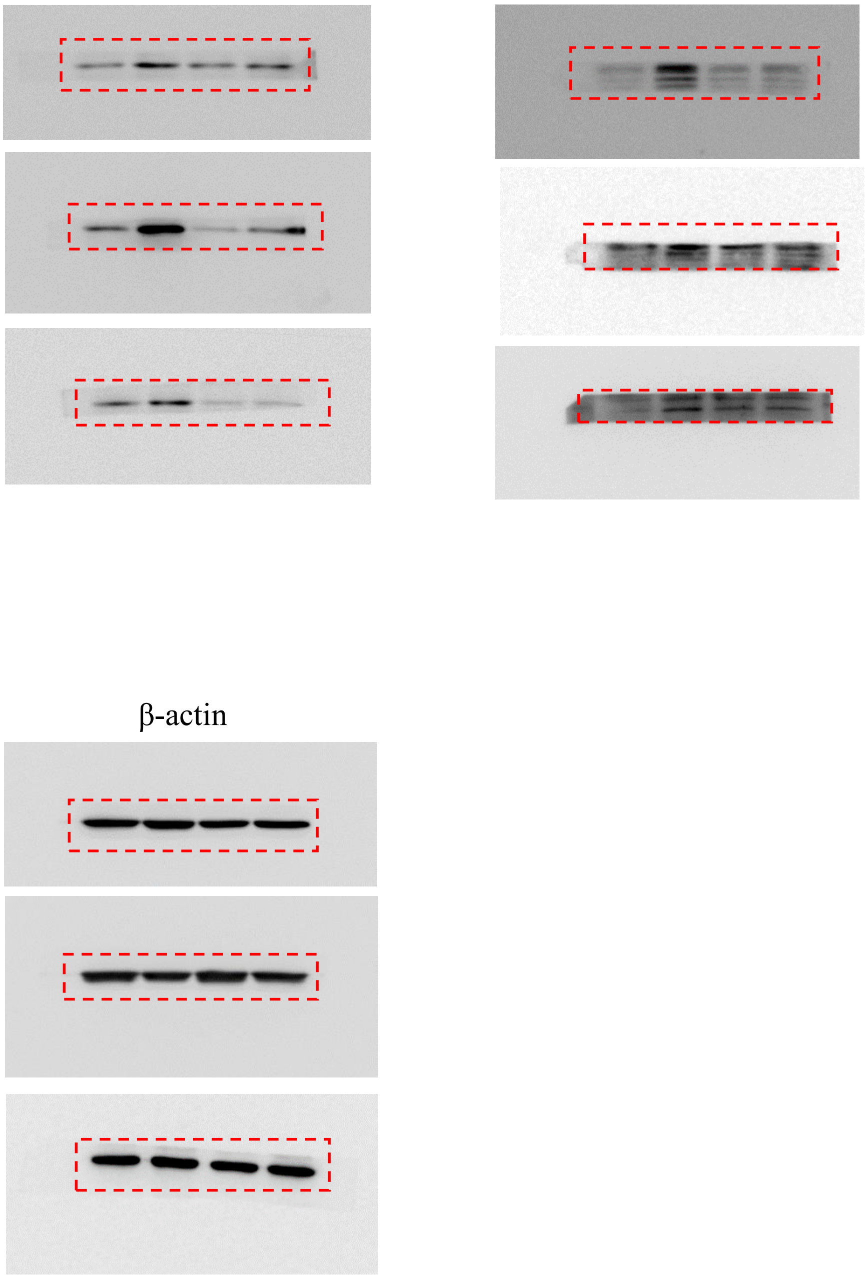

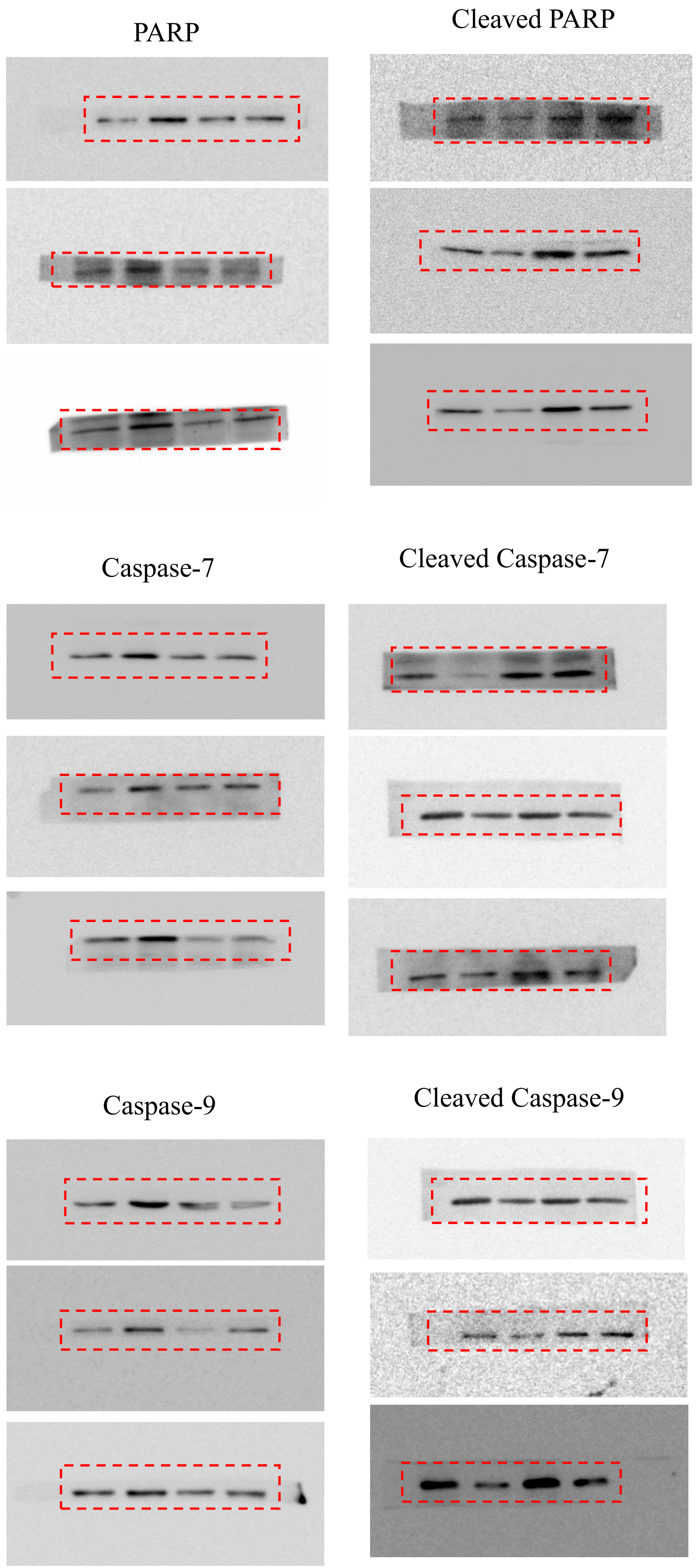
PARP
Cleaved PARP
Caspase-7
Cleaved Caspase-7
Caspase-9
Cleaved Caspase-9

Fig.4E

G6PD

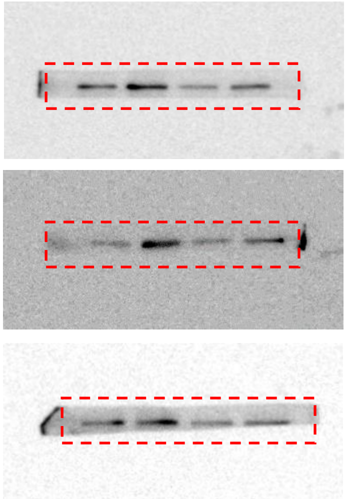

Bcl-2

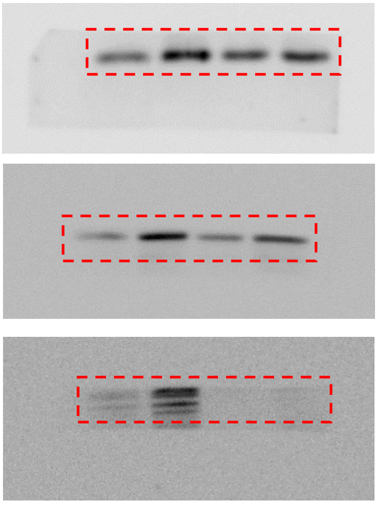

β-actin

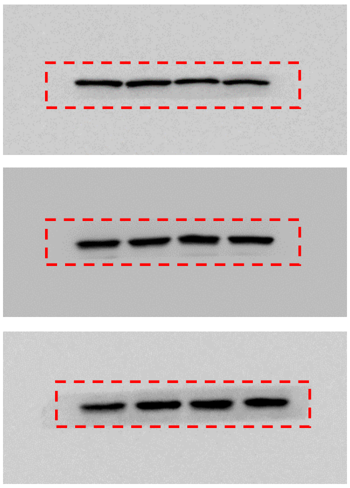

PARP

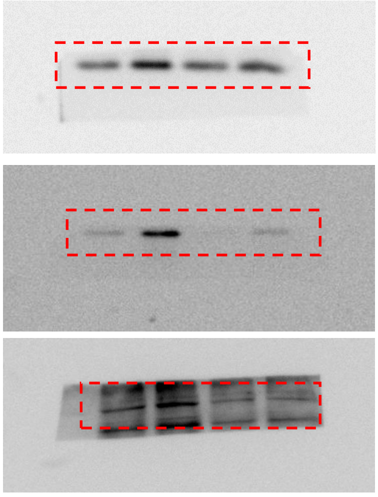

Cleaved PARP

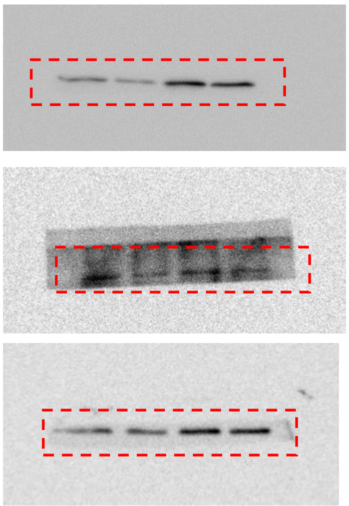

Caspase7

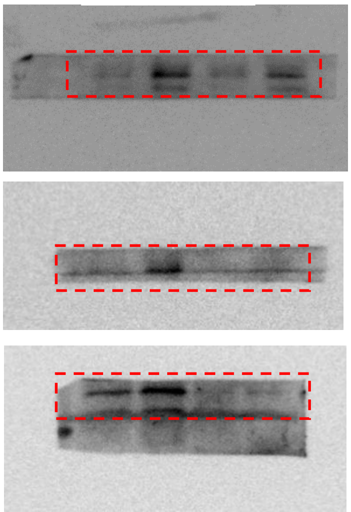

Cleaved Caspase-7

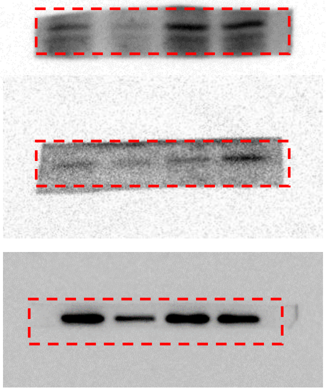

Caspase-9

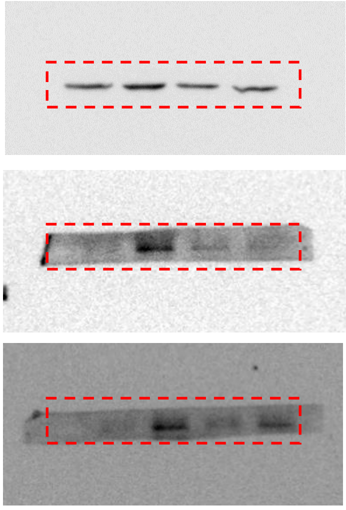

Cleaved Caspase-9

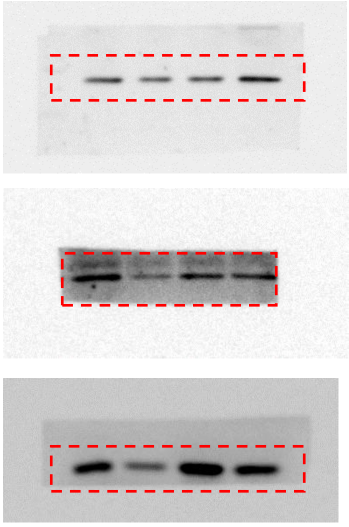

Fig.5E

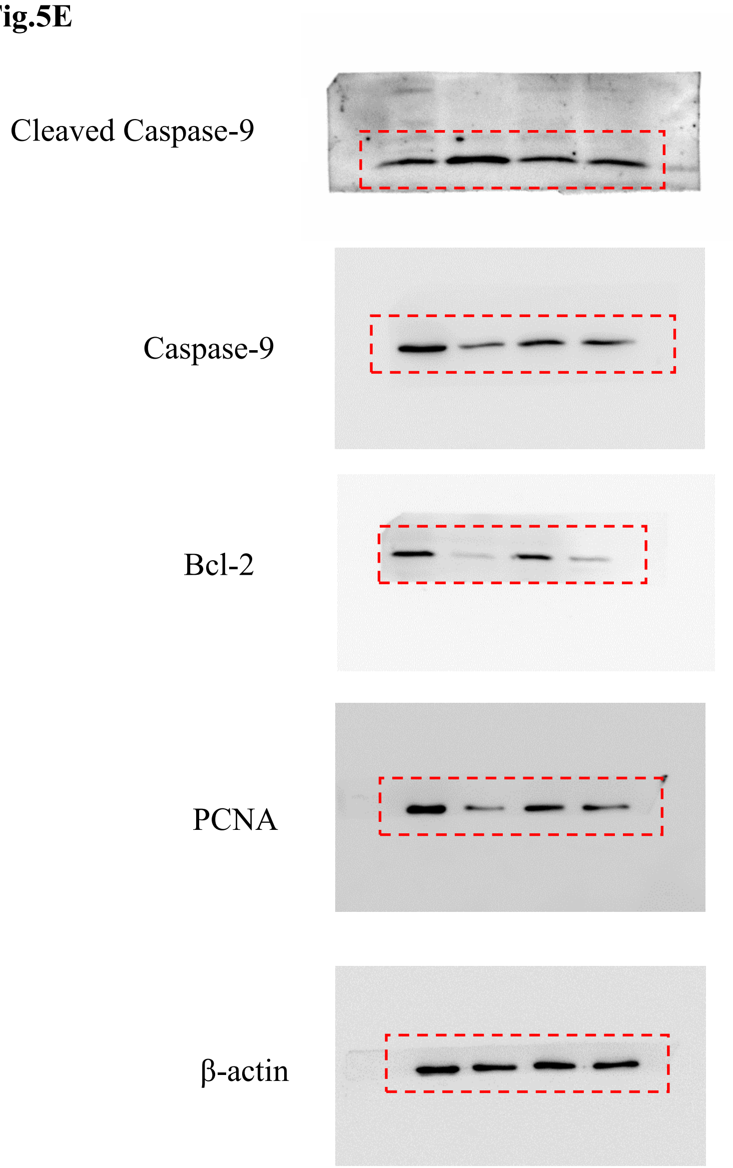
Cleaved Caspase-9
Caspase-9
Bcl-2
PCNA
β-actin

Fig .6A

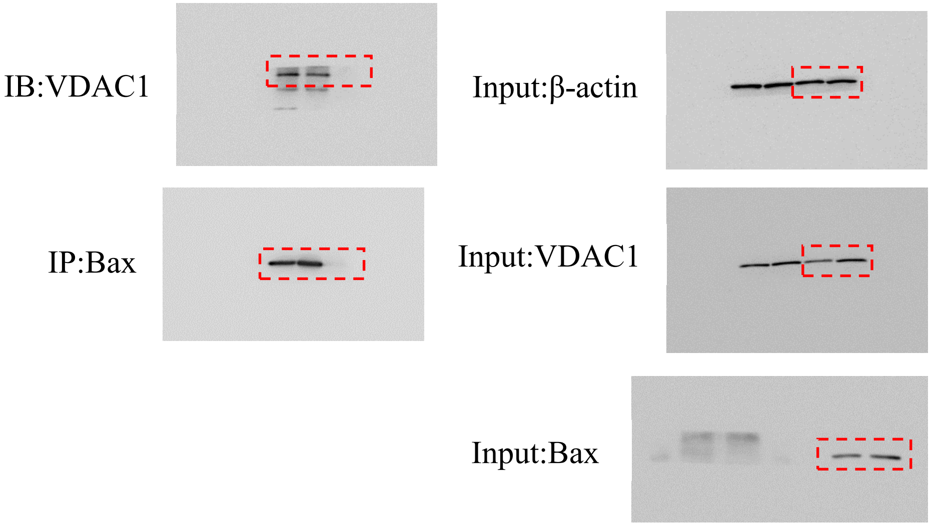
IB:VDAC1
Input:β-actin
IP:Bax
Input:VDAC1
Input:Bax

Fig .6B

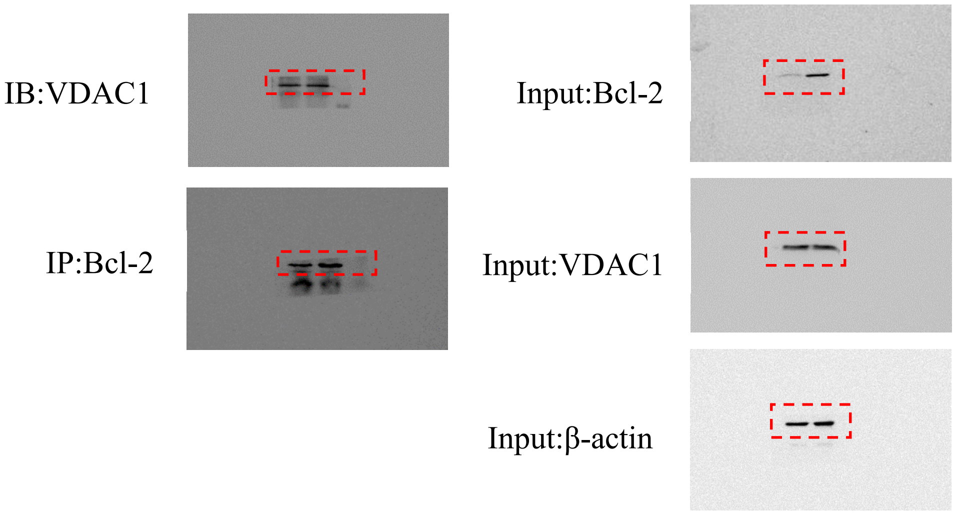
IB:VDAC1
Input:Bcl-2
IP:Bcl-2
Input:VDAC1
Input:β-actin

Fig .6C

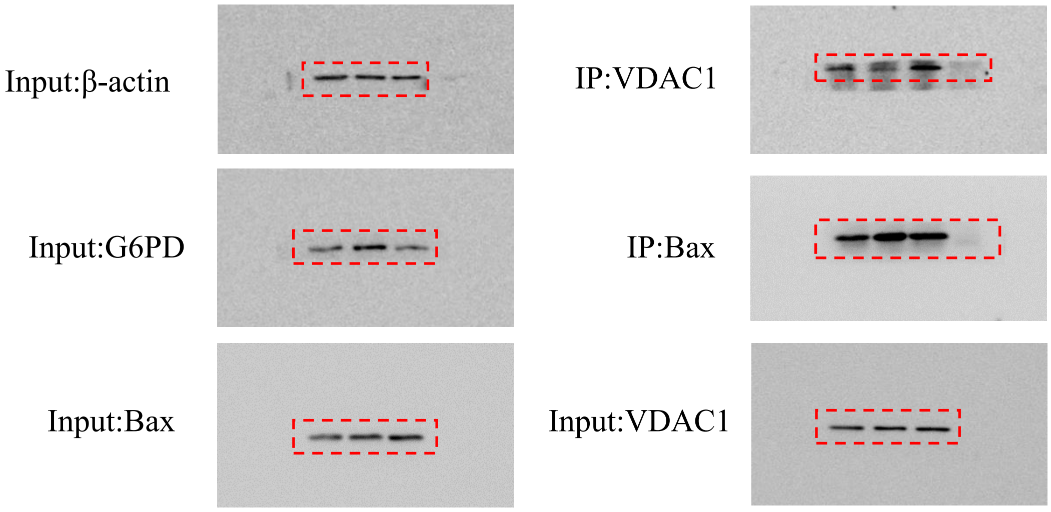
Input:β-actin
IP:VDAC1
Input:G6PD
IP:Bax
Input:Bax
Input:VDAC1

Fig .6D

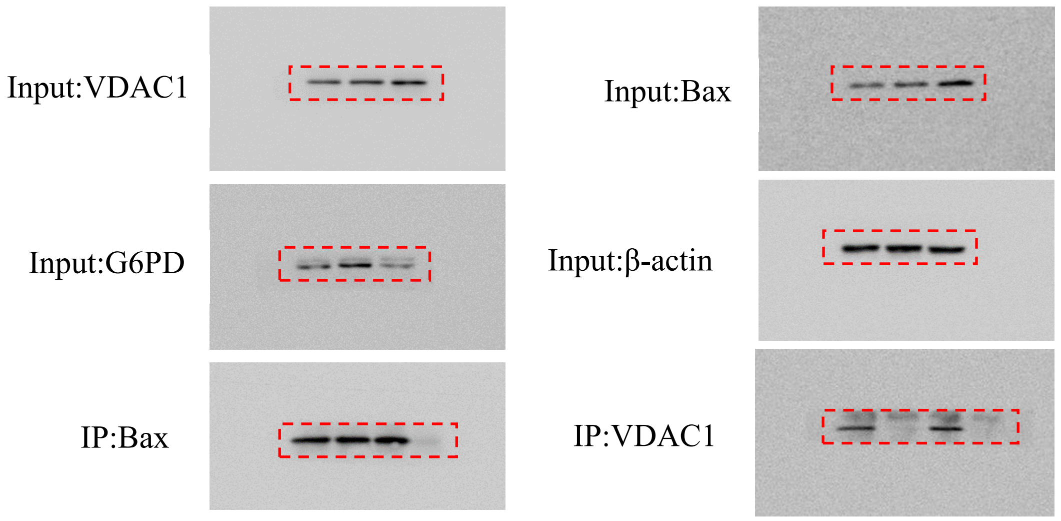
Input:VDAC1
Input:Bax
Input:G6PD
Input:β-actin
IP:Bax
IP:VDAC1

Fig .6E

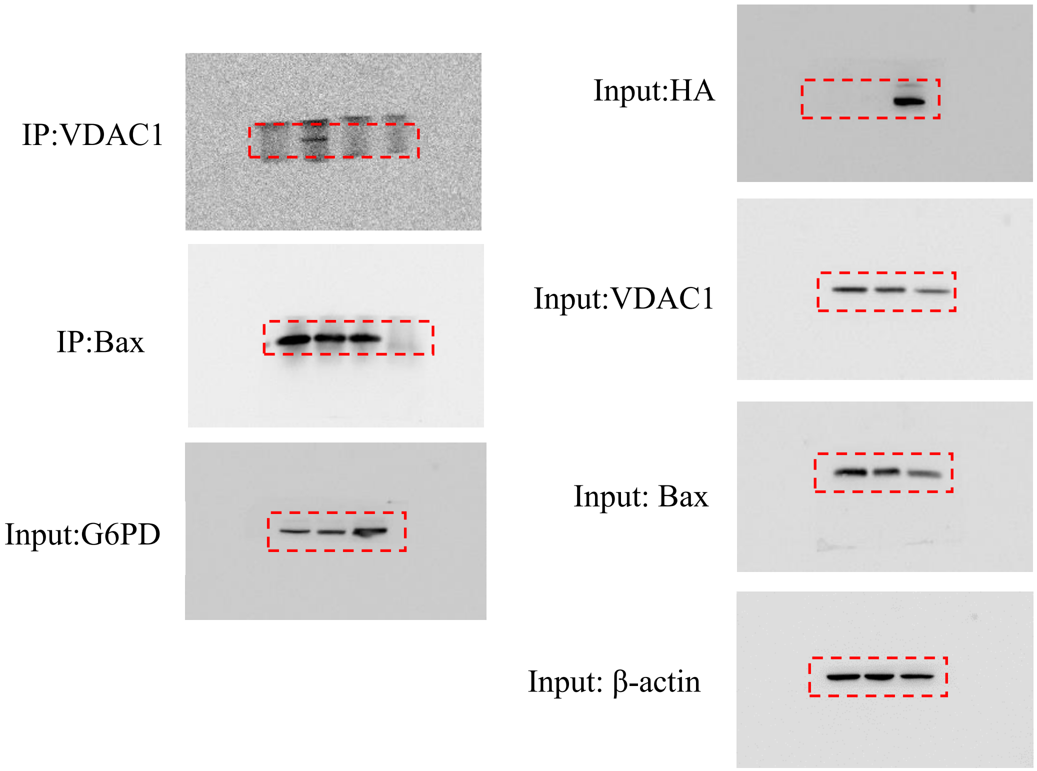
Input:HA
IP:VDAC1
IP:Bax
Input:VDAC1
Input: Bax
Input:G6PD
Input: β-actin

Fig .6F

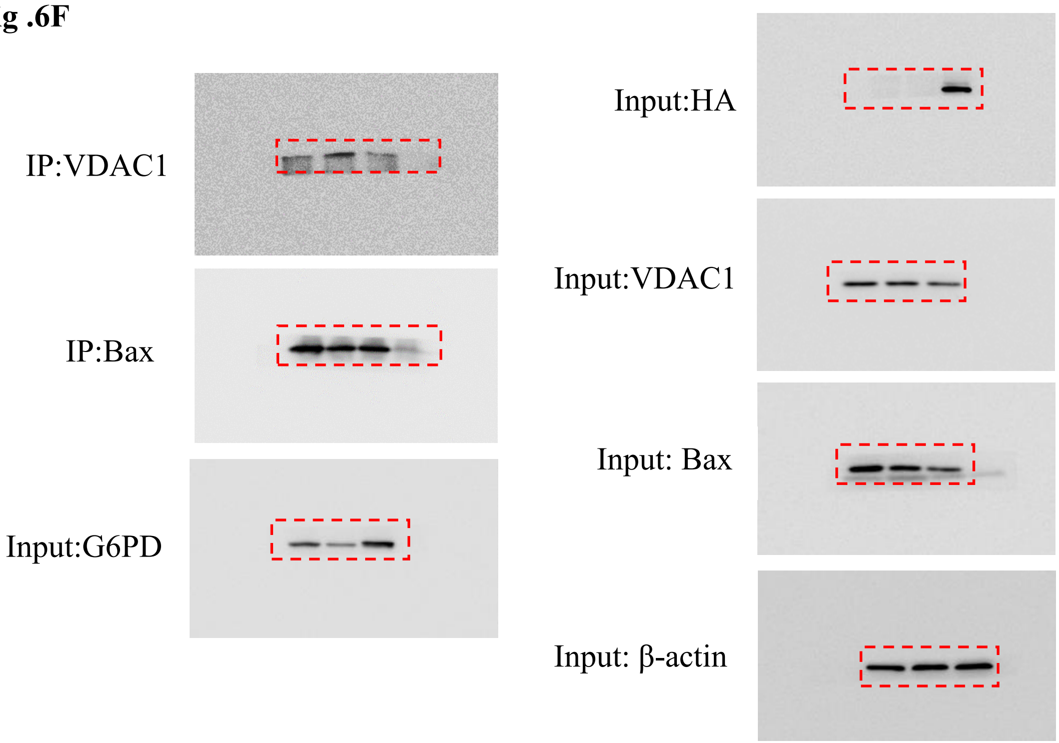

Fig .6G

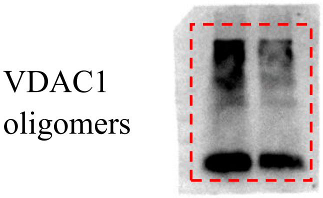

Fig .6H

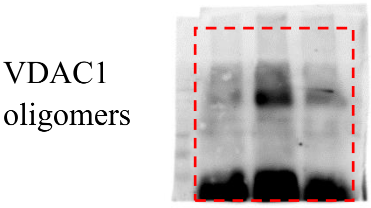

Fig .6I

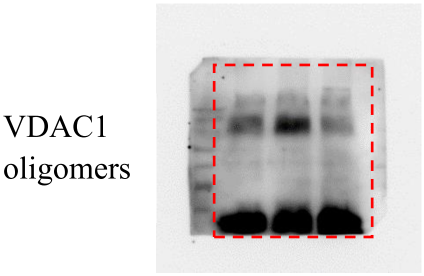

Fig.S1A

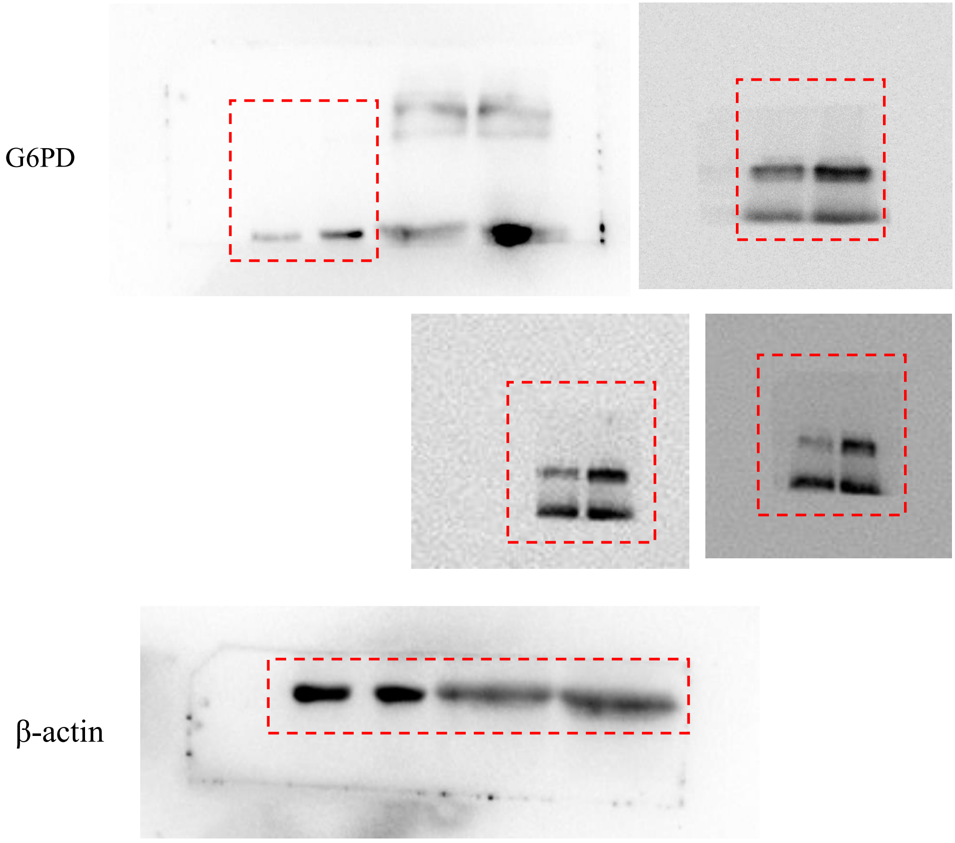

Fig.S1C

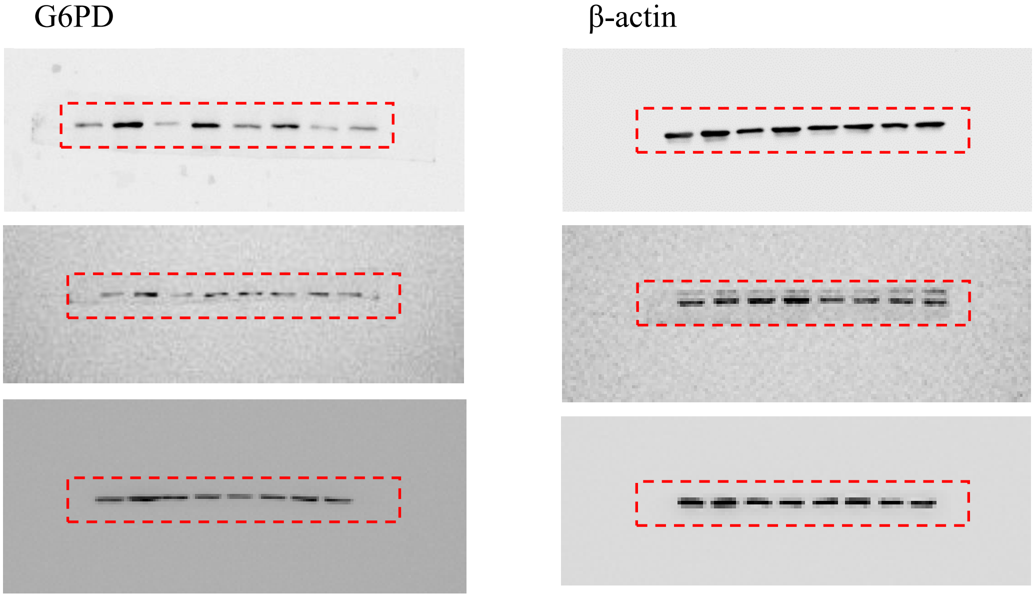

Fig.S1D

G6PD

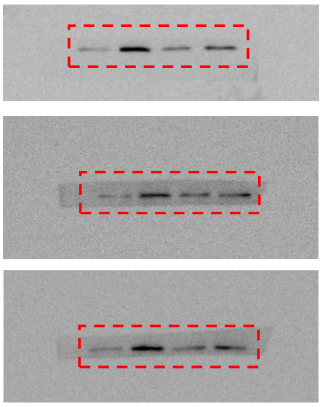

PCNA

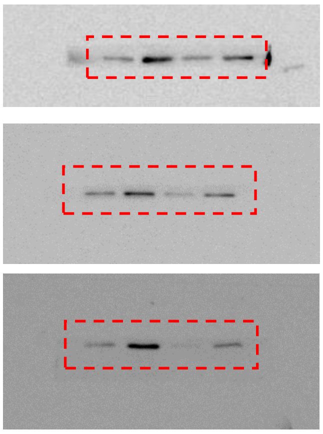

SM22α

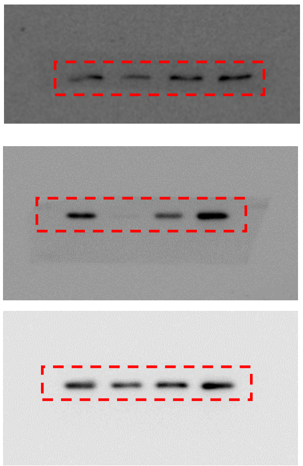

β-actin

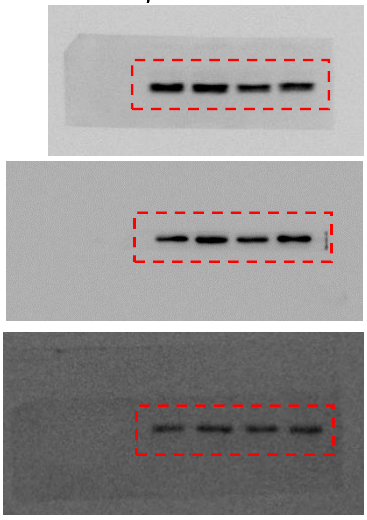

α-actin

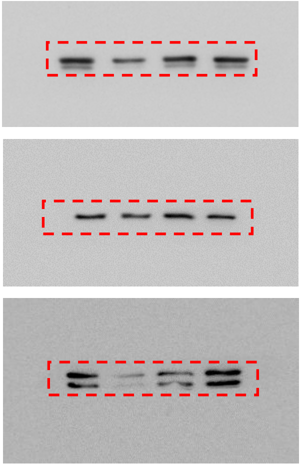

Fig.S3B

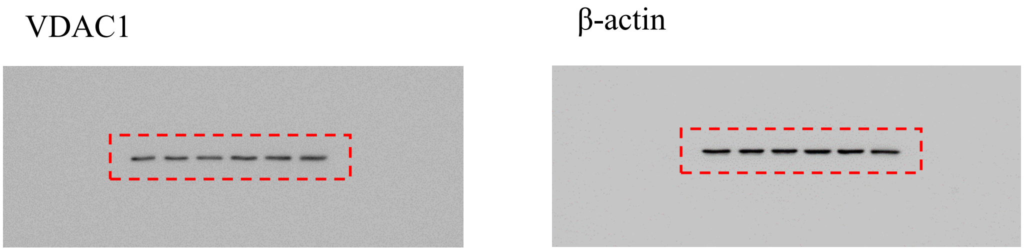

Fig.S3C

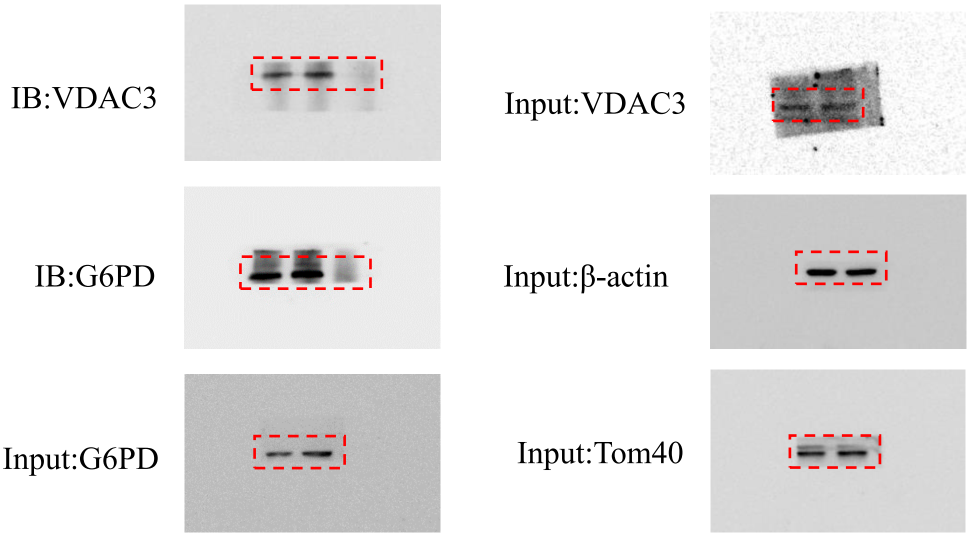

Fig.S3D

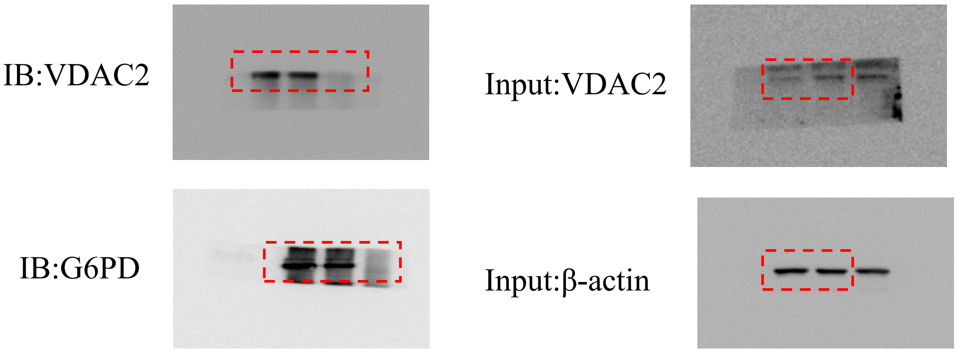

Fig.5E

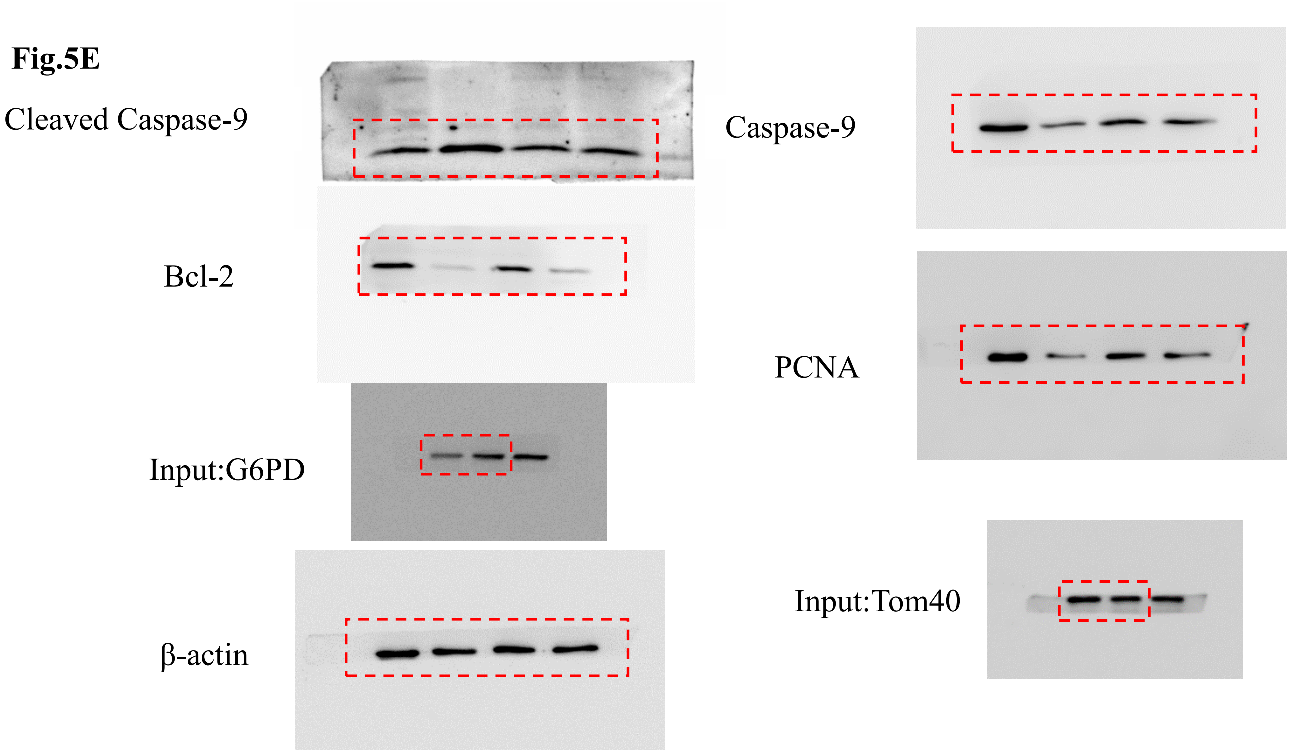

Fig.S3E

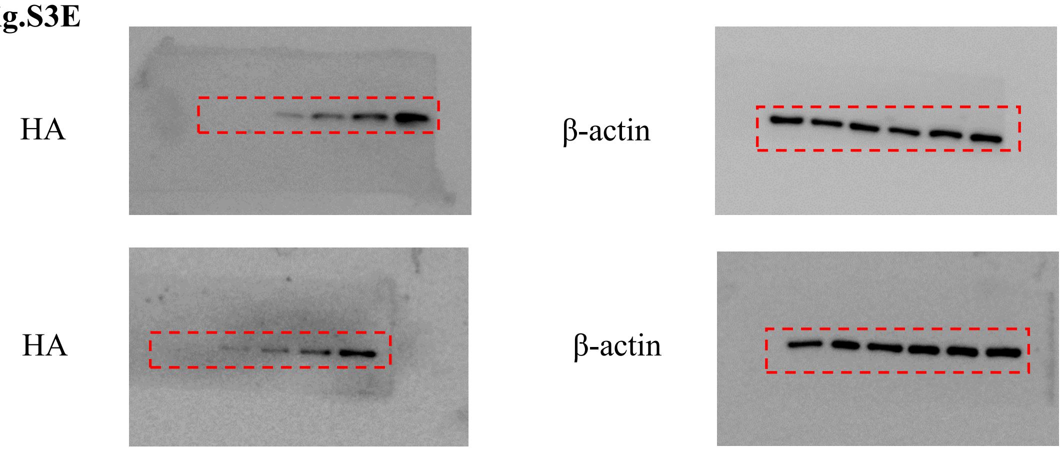

Fig.S4A

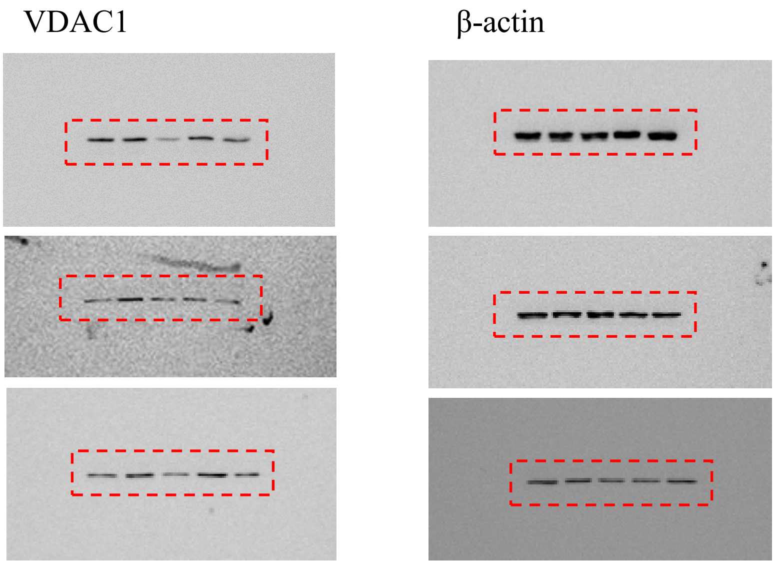

Fig.S4B

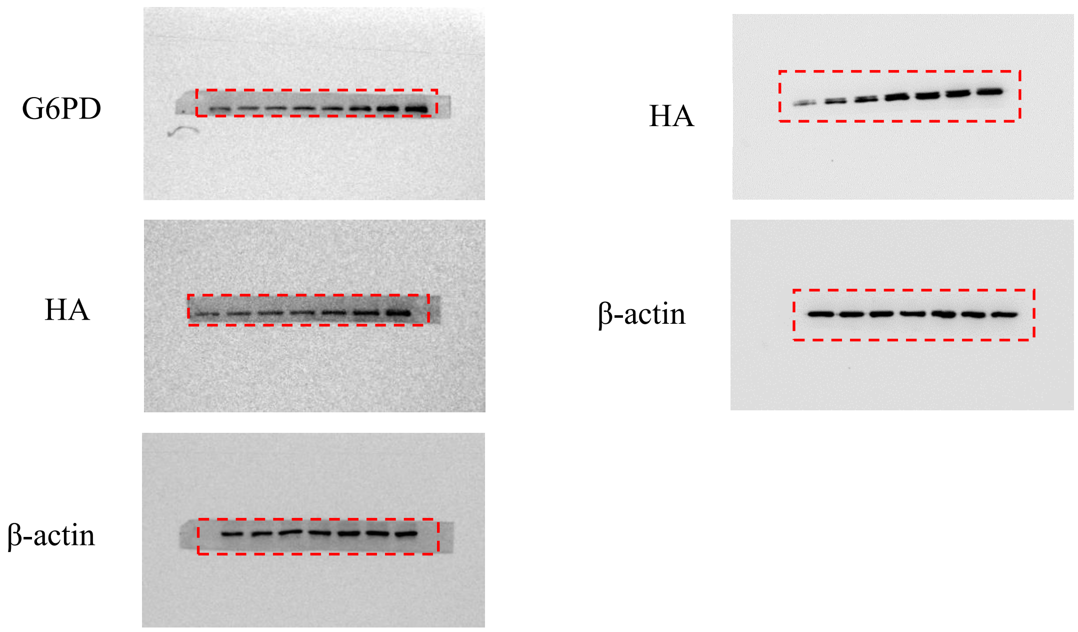

Fig.S4C

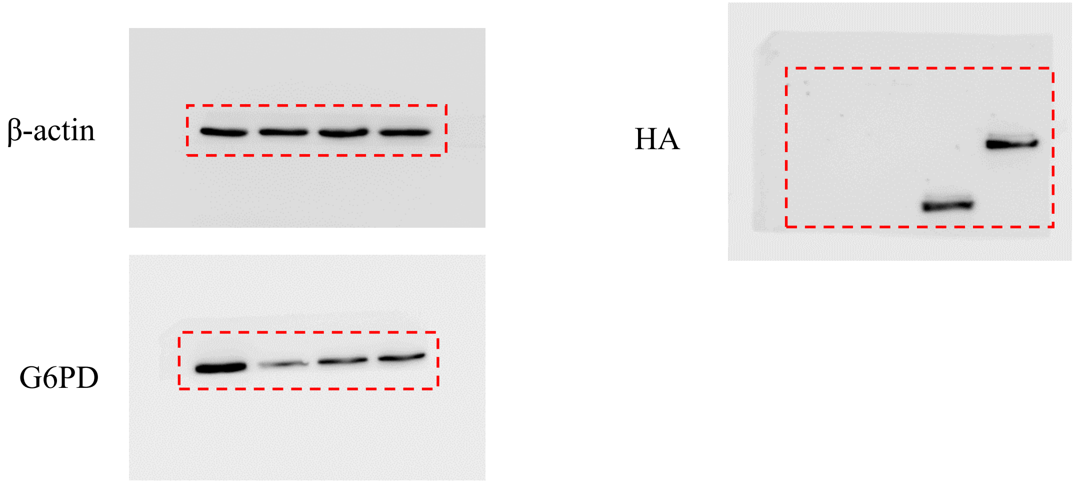

Supplement: Supplementary file 3 — Additional file 3. Original western blots. [file 11658_2024_566_MOESM3_ESM.pdf]
